# YAP/TAZ activation predicts clinical outcomes in mesothelioma and is conserved in in vitro model of driver mutations

**DOI:** 10.1002/ctm2.1190

**Published:** 2023-02-05

**Authors:** Richard Cunningham, Siyang Jia, Krishna Purohit, Omar Salem, Ning Sze Hui, Yue Lin, Neil O. Carragher, Carsten Gram Hansen

**Affiliations:** ^1^ Centre for Inflammation Research Institute for Regeneration and Repair Edinburgh BioQuarter University of Edinburgh Edinburgh UK; ^2^ Cancer Research UK Scotland Centre Institute of Genetics and Cancer University of Edinburgh Edinburgh UK

**Keywords:** YAP/TAZ, stratification, mesothelioma, BAP1, NF2

## Abstract

The Hippo signalling pathway is dysregulated across a wide range of cancer types and, although driver mutations that directly affect the core Hippo components are rare, a handful is found within pleural mesothelioma (PM). PM is a deadly disease of the lining of the lung caused by asbestos exposure. By pooling the largest‐scale clinical datasets publicly available, we here interrogate associations between the most prevalent driver mutations within PM and Hippo pathway disruption in patients, while assessing correlations with a variety of clinical markers. This analysis reveals a consistent worse outcome in patients exhibiting transcriptional markers of YAP/TAZ activation, pointing to the potential of leveraging Hippo pathway transcriptional activation status as a metric by which patients may be meaningfully stratified. Preclinical models recapitulating disease are transformative in order to develop new therapeutic strategies. We here establish an isogenic cell‐line model of PM, which represents the most frequently mutated genes and which faithfully recapitulates the molecular features of clinical PM. This preclinical model is developed to probe the molecular basis by which the Hippo pathway and key driver mutations affect cancer initiation and progression. Implementing this approach, we reveal the role of NF2 as a mechanosensory component of the Hippo pathway in mesothelial cells. Cellular NF2 loss upon physiological stiffnesses analogous to the tumour niche drive YAP/TAZ‐dependent anchorage‐independent growth. Consequently, the development and characterisation of this cellular model provide a unique resource to obtain molecular insights into the disease and progress new drug discovery programs together with future stratification of PM patients.

## Graphical abstract

1. Stratifying pleural mesothelioma (PM) patients reveals active YAP/TAZ is associated with poor clinical outcomes.
2. An isogenic cell‐line model of PM driver mutations is developed that faithfully recapitulates clinical PM characteristics.
3. NF2 regulates YAP/TAZ mesothelial activity in response to stresses, including sensing mechanical cues, while BAP1 appears to regulate a stem‐cell‐like transcriptional program.

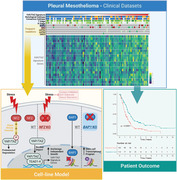

## 1 INTRODUCTION

Pleural mesothelioma (PM) is a rare cancer of the mesothelial pleural lining of the lung, most commonly associated with exposure to the carcinogen asbestos. PM is most common in males, likely due to more frequent exposure to asbestos fibres.^1^, ^2^, ^3^ Despite the declining use of asbestos as an insulating and fire retardant agent in the developed world,^4^ the prevalent presence of asbestos in buildings and the overall latency of upwards of 30 years from exposure to when mesothelioma presents clinically means that rates of PM have continued to increase. The peak incidence of PM is projected to occur within the next decade.^3^, ^5^, ^6^, ^7^ Consequently, the continued mining of asbestos and underdiagnosis of PM in the developing countries,^8^, ^9^ together with the realisation that some types of currently used nanofibers cause PM‐like cancers in rodent models,^10^ strongly indicate that PM is a disease with a continued unmet clinical need. PM is characterised by a low mutational burden.^11^, ^12^, ^13^ Patients who are diagnosed have a poor prognosis with survival rates one of the lowest of any cancer, while the current standard of care treatments extend survival by a matter of months.^7^ Some progress has been made in recent years, with the successful combination of nivolumab and ipilimumab showing potential in extending median overall survival in a small subgroup of patients by up to 4 months.^14^ However, current treatments are essentially palliative care.^15^ As such, the search for a curative therapeutic regime remains elusive, highlighting the pressing need for the development of effective therapeutics to enable clinical management of the disease.

The difficulties in identifying effective therapeutics to manage PM are caused by late‐stage diagnosis and an infiltrative and therefore overall malignant phenotype. In addition, there is currently a lack of predictive preclinical models which inform disease positioning/patient stratification. An in‐depth understanding of the initial molecular drivers, as well as the complicit oncogenic pathways that facilitate the progression of this refractory cancer type, are consequently needed.^16^, ^17^ To this end, a number of studies have, through whole exome sequencing, examined in detail the molecular landscape of PM,^12^, ^13^, ^18^ describing genomic and transcriptomic alterations most closely aligned with mesothelioma onset and initiation. These analyses have revealed that a number of loss‐of‐function mutations present at low frequency in other cancer types are frequently identified during the development of PM, with two tumour suppressors most notably lost regularly in PM patients: *BAP1*, which acts as a deubiquitinase^19^, ^20^ and *NF2*, a major upstream activator of the core kinase module of the Hippo signalling pathway.^21^, ^22^ Beyond *NF2*, a number of additional key upstream regulators of the Hippo pathway are inactivated at lower frequencies in PM.^12^, ^13^

The Hippo pathway consists of an upstream serine/threonine kinase module that when active, phosphorylates and thereby inhibits the transcriptional co‐activators YAP and TAZ, comprising the transcriptional module of the pathway. When YAP and TAZ are unphosphorylated, they localise to the nucleus and bind to TEAD family transcription factors, driving the expression of target genes.^23^, ^24^, ^25^ The loss‐of‐function mutations in the upstream regulatory module of the Hippo pathway are consequently of relevance, as the loss of components within this kinase module is predicted to cause hyperactive YAP/TAZ. In other cancers and cell model systems, YAP and TAZ drive EMT,^26^, ^27^, ^28^ migration/metastasis,^29^, ^30^ and chemoresistance,^31^, ^32^ while their expression and activation are associated with poor clinical outcomes across a range of cancer types.^33^, ^34^, ^35^, ^36^ This body of work reported here describes an integrated analysis of the largest scale studies of PM patients to date.

*BAP1* and *NF2* are the two most commonly mutated genes in PM and are loss‐of‐function mutated in up to 57% and 23% of cases respectively.^12^, ^13^, ^18^, ^37^ Mutation status within these genes, when considered in combination, represents a powerful prognostic indicator of PM^38^; with the push towards realising precision oncology well underway, a sensible question is whether populations grouped according to the mutation status of these drivers might represent actionable subtypes within PM. Our work describes a pooled, detailed analysis of high‐throughput PM datasets^12^, ^13^ to explore potential stratification approaches. This approach allows us to investigate the link between the major driver mutations in PM and the Hippo signalling pathway. We additionally develop and characterise a pre‐clinical in vitro cellular model of PM that allows for follow‐up investigations into the dynamics and differential effects of the Hippo pathway and BAP1 dysregulation in PM.

## 2 RESULTS

### 2.1 Interrogating large‐scale databases to define patient subgroups in PM

**FIGURE 1:**
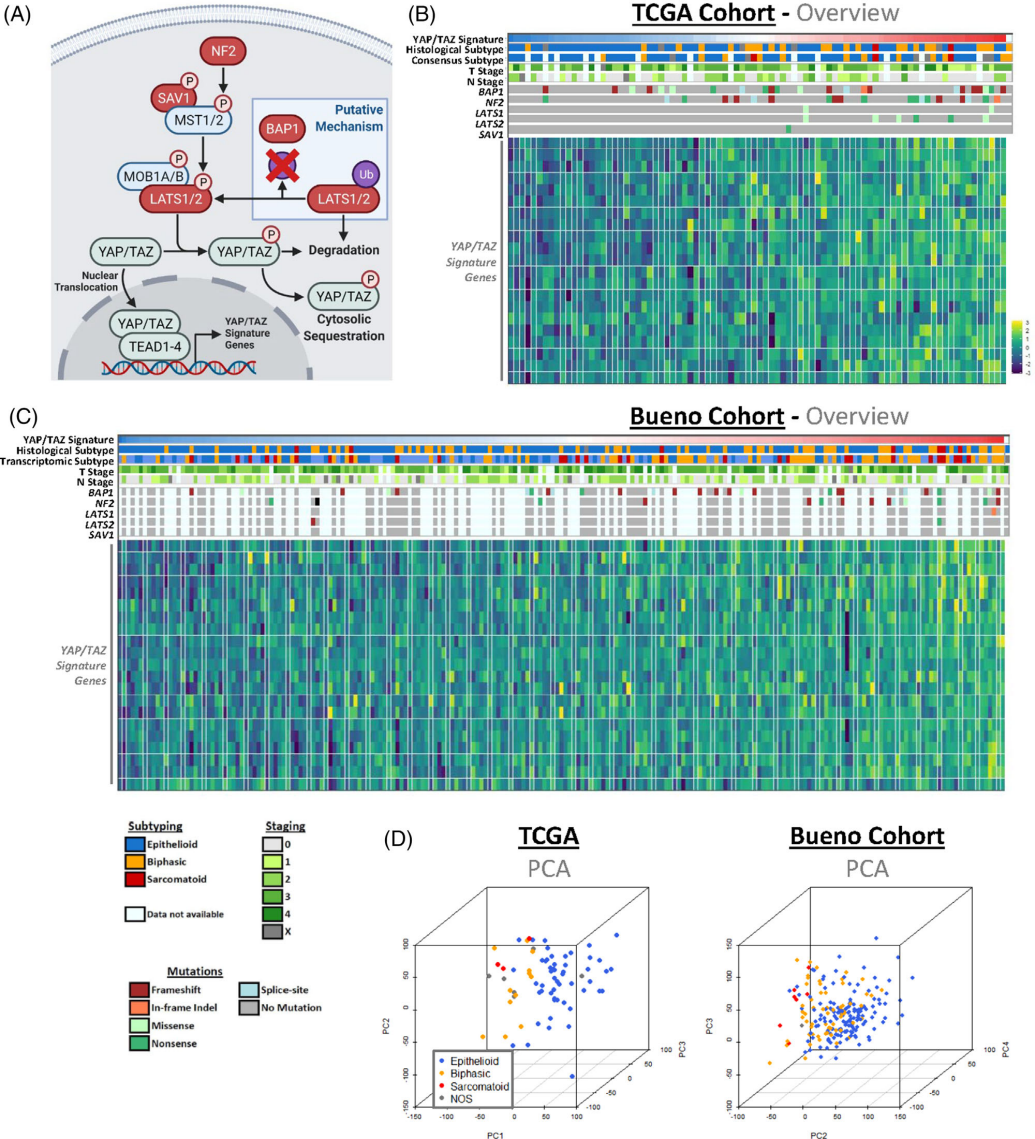
Defining distinct populations of pleural mesothelioma patients. (A) Schematic shows key components of the Hippo signalling pathway, alongside the suggested role BAP1 plays in its regulation (blue box). Elements reported as mutated in cases of pleural mesothelioma (PM) are highlighted in red. (B) Heatmap highlights the various clinical, genomic and transcriptomic features of the The Cancer Genome Atlas (TCGA) cohort of 87 PM patients.^12^ Patients are ordered in ascending gene set variation analysis (GSVA) scoring of YAP/TAZ signature gene expression,^40^ with the expression of individual genes also shown (bottom). Clinical outputs (top) include histological subtyping classifications, both original and as determined via consensus pathology review, and American Joint Committee on Cancer (AJCC) staging, while predicted Hippo kinase cascade inactivating mutation status (centre) are highlighted according to variant classification, with mutations in *NF2*, *LATS1/2* and *SAV1* included. (C) Heatmap, as in (B), details 212 patients constituting the Bueno et al.^13^ cohort. Legend for both heatmaps (B, C), highlighting colour schema for subtyping, mutation status, and clinical staging of PM cases, shown below. (D) Principal component analysis (PCA) plots, depicted in 3D, show patients coloured according to histological subtype, with dimensionality reduction applied to full transcriptomes. This analysis highlights that broad transcriptional profiling is sufficient to distinguish between the different histological subtypes in both the consensus subtypes defined in the TCGA (left; *n* = 86) and Bueno et al. (right; *n* = 211) cohorts

This work describes the combination of two large PM databases, the first of which was generated by The Cancer Genome Atlas (TCGA),^12^ consisting of 87 patients and the second of which includes 211 patients^13^ (hereon referred to as TCGA cohort and Bueno cohort, respectively). As the occurrence of inactivating mutations in the upstream regulatory kinase module of the Hippo pathway is emerging as a common phenomenon associated with the development of PM,^39^, ^40^ patients were initially characterised according to Hippo pathway activation status. This was approached by initially assessing the mutation status of key Hippo pathway players, regularly identified in PM patients (Figure 1A). Secondly, the activity of YAP/TAZ, the transcriptional co‐activators within the downstream arm of the Hippo pathway, was approximated using a previously defined, curated gene set of bona fide YAP/TAZ transcriptional targets.^41^

Assessing these various metrics, the broad make‐up of both datasets is consistent and both patient cohorts across datasets are directly comparable (Figure 1B,C), with no over‐representation of subtype/clinical stage or mutations in either. Principal component analysis (PCA) of patient transcriptomes grouped by histological subtype reveals a general clustering of subtype groups (Figure 1D and Figure S1A), which points to the potential of broad transcriptional profiles to predict simple clinical outputs. In order to explore patient stratification, various clinical outputs were assessed upon the grouping of patients according to the mutation status of two key driver mutations within PM: *NF2* and *BAP1*. Interestingly, although these genes are frequently loss‐of‐function mutated in cases of PM, no single distinct mutation is common amongst patients. Considering specific alterations in amino acids of protein products, two patients exhibited aberrant stop codons at both arginine 57 and tyrosine 153 in NF2, while two patients each harboured serine 10 to arginine, cysteine 91 to glycine, and asparagine 645 to lysine, while all other residue shifts in NF2 and BAP1 were unique. Interestingly, two, 13 and 13 out of 36 impactful *NF2* mutations include missense mutations, nonsense mutations and frameshift indels respectively, while out of 54 impactful *BAP1* mutations, 9, 10, and 25 were missense mutations, nonsense mutations, and frameshift indels respectively. The remainder of the mutations include in‐frame indels and splice‐site mutations, indicating a high frequency of highly disruptive genomic perturbations within these driver genes. When grouping patients according to the mutation status of these drivers, initial PCA highlights that patient groups do not readily cluster according to broad transcriptional profiles (Figure S1B). Further analyses reveal that there are no significant associations between mutation status and transcriptomic subtypes (Figure S1C), a molecular subgrouping approach which mirrors histological subtypes,^13^ clinical stages (Figure S1D), or overall patient survival (Figure S1E). The absence of any transcriptomic clustering or clear association with prognostic indicators suggests that profiling patients according to mutation status may not be meaningful clinically; however, in spite of the lack of significance, trends suggest that *BAP1* mutation may loosely associate with earlier‐stage PM and the epithelioid subtype, an observation which is corroborated by previous studies.^42^, ^43^, ^44^, ^45^

### 2.2 YAP/TAZ activation status predicts prognosis in PM patients

Notably, *NF2* is the most frequently mutated Hippo pathway component in PM, however, multiple key members of the upstream core kinase cascade (*LATS1*, *LATS2*, *SAV1*), are also loss‐of‐function mutated^13^, ^18^, ^39^ (Figure 1A–C). These observations suggest that Hippo kinase cascade inactivation (and therefore YAP/TAZ activation) likely plays a major role in PM onset, while both NF2 (also known as Merlin) and BAP1 have been tied to the Hippo pathway as a bona fide key regulator^46^, ^47^ and a putative effector of core kinase members,^48^ respectively. If the loss of either *NF2* or *BAP1* mediates oncogenesis via the perturbation of Hippo signalling, the failure to take into account the additional Hippo kinase module inactivating events present in PM may have confounded previous efforts to stratify patients according to mutation status. Collectively, these observations suggest that subgrouping patients according to YAP/TAZ activation status may be a more biologically meaningful stratification strategy.

**FIGURE 2:**
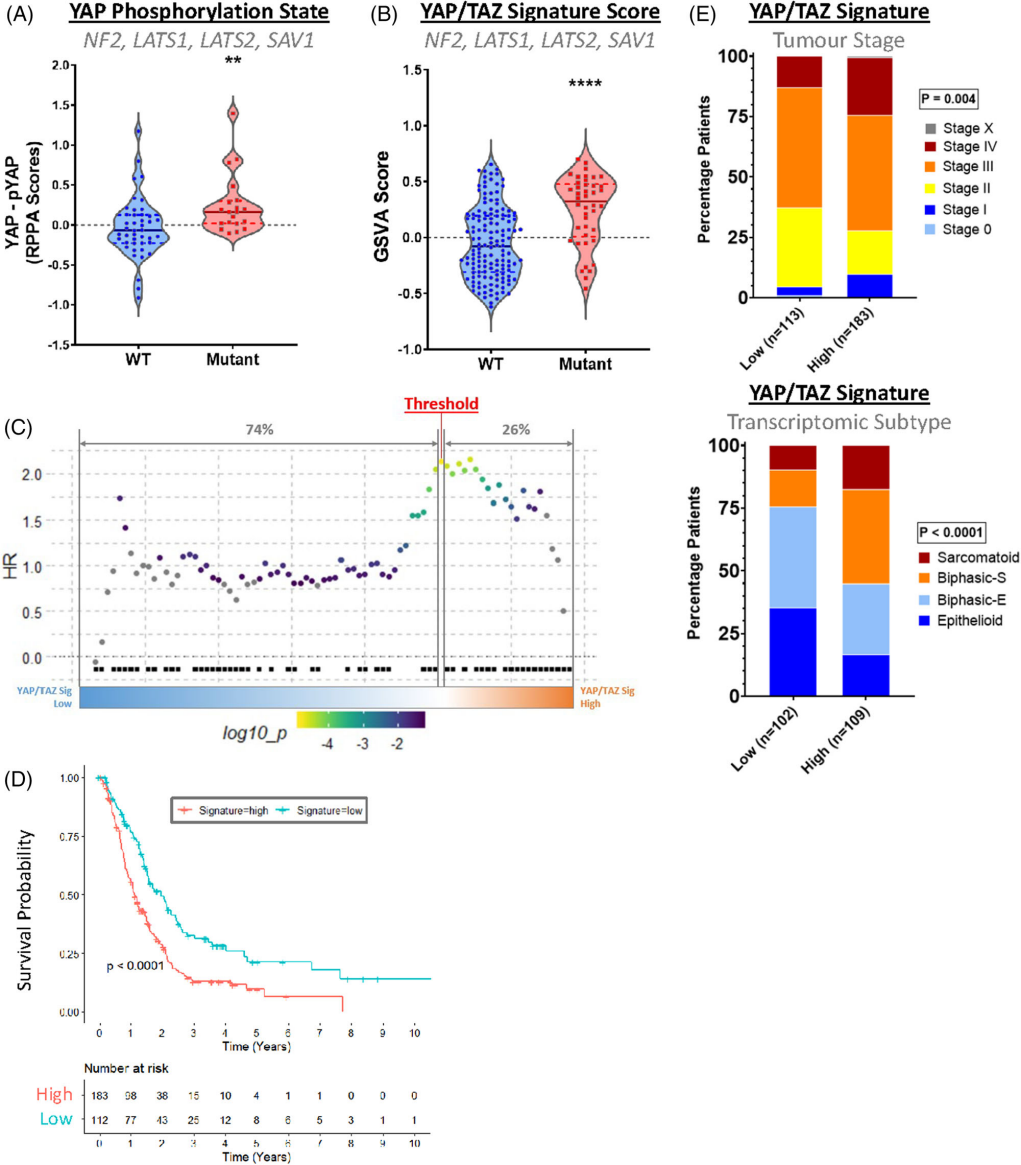
Expression of YAP/TAZ signature genes correlates with poor clinical outcomes in pleural mesothelioma (PM) patients. (A) Violin plot shows relative levels of unphosphorylated YAP in patient material, thereby approximating activity. This was calculated by subtracting levels of YAP phosphorylated at serine 127, an inhibitory post‐translational modification, from total YAP protein levels, recorded in reverse phase protein array (RPPA) data in The Cancer Genome Atlas (TCGA) dataset (*n* = 63). The mutant grouping includes Hippo pathway components *NF2*, *LATS1*, *LATS2* and *SAV1*. (B) Violin plot shows gene set variation analysis (GSVA) scores of YAP/TAZ signature gene expression in patients split according to Hippo kinase cascade inactivating mutation status. A significant collective overexpression of these genes is observed in patients with mutations in genes associated with Hippo signalling when both cohorts are combined (*n* = 184). (C) SurvivALL plot shows the chosen methodology of setting thresholds based on the YAP/TAZ signature. Patients from the TCGA cohort are ordered according to the increasing signature, as determined by GSVA scores, with hazard ratios (HRs) for all possible thresholds shown as a scatterplot. Deaths are denoted by black squares, while the scale shows the colouring of points according to HRs. The thresholding that yields the highest HR is highlighted (red) and the percentage of patients classed as signature low and signature high are shown above (grey). (D) Kaplan‐Meier curve shows the overall survival of patients split according to YAP/TAZ signature thresholds. Patients classed as YAP/TAZ signature high experience a reduction in overall survival when both datasets are grouped. HR = 1.75, *n* = 296, the threshold set at 38%. (E) Barplots show T staging (top) in grouped datasets and consensus subtype (bottom) in the Bueno et al. cohort (*n* = 211, threshold set at 52%), with patients, split according to YAP/TAZ signature. Patients categorised as signature high generally exhibit more advanced clinical stages and more aggressive subtypes. *p‐*Values in (A) and (B) were determined by the Mann‐Whitney U test, *p‐*value and HR for (D) were calculated via log‐rank test and Cox proportional hazard model respectively, while *p‐*values for (E) were calculated via Fisher's exact test. **p < .01 and ****p < .0001 relative to wild‐type (WT)

Upregulation of *YAP1* and *WWTR1*, the genes that encode YAP and TAZ respectively, have previously been used as a marker of Hippo pathway inactivation,^49^, ^50^ however this is an indirect measure, as YAP/TAZ are predominantly regulated via protein levels and subcellular location.^51^, ^52^ LATS1/2 mediated phosphorylation of YAP and TAZ leads to the cytoplasmic sequestering of these two transcriptional co‐activators, whereas unphosphorylated YAP and TAZ translocate to the nucleus and bind to TEAD family transcription factors (Figure 1A).^51^, ^52^ Within the patient cohorts, it is possible to approximate a more direct metric of activity via quantification of levels of total YAP relative to phosphorylated, inactive YAP at the protein level.^53^ Utilising this approach, protein quantification within tumour samples in the TCGA cohort^12^ via reverse phase protein array (RPPA) reveals that presumed active YAP levels are higher in patients affected with any common, canonical Hippo kinase module inactivating mutation (*NF2*, *LATS1*/*2*, *SAV1*) (Figure 2A). As further validation of this inferred approach for the transcriptional Hippo pathway activity within this subgrouping, the collective expression of YAP/TAZ downstream targets, as defined by TCGA,^41^ were assessed. This 22‐gene signature is comprised of target genes associated with a variety of cellular functions, including two primary markers of YAP/TAZ activity *CCN1* (or *CYR61*) and *CCN2* (or *CTGF*), with gene‐set expression quantified via gene set variation analysis (GSVA), allowing a single signature metric for each patient. This analysis shows a robust upregulation of the YAP/TAZ signature within patients harbouring a canonical Hippo kinase module inactivating mutation, both in each individual patient cohort (Figure S2A) and upon pooling all patients into a single dataset (Figure 2B). Although BAP1 is not part of the Hippo pathway, a recent report highlights that BAP1 functions as a stabiliser of LATS1 and LATS2 via its deubiquitinase activity in a pancreatic cancer cell‐line model, hence acting as an activator of the Hippo kinase module (Figure 1A).^48^ Loss of BAP1 in PM is associated with a slight increase in expression of YAP/TAZ targets in patients, though this is not conserved in both patient cohorts (Figure S2B).

With a reliable metric for determining Hippo kinase module inactivation established, it is possible to consider an effective strategy for stratifying patients meaningfully according to YAP/TAZ activity. Conventionally, subgrouping patients according to a continuous variable involves setting arbitrary thresholds, typically splitting patients around a median score. While a commonly employed approach, this is not necessarily relevant, as it fails to take sufficiently into account the biology surrounding a phenomenon. As a means to address this shortcoming, we employed SurvivAll,^54^ a package that maximises the hazard ratio of high/low subgroups via an iterative grouping process (Figure 2C). Applying the resulting subgroups to the pooled dataset reveals a significant and dramatic decrease in median survival in YAP/TAZ high vs low patients (median survival 13.32 months vs. 23.52 months) (Figure 2D). Additionally, YAP/TAZ activity is found to be increased in both late‐stage PM and the aggressive sarcomatoid subtype (Figure 2E). These results are furthermore consistent between both cohorts when assessed separately (Figure S2C,D), validating the observation that active YAP/TAZ is a predictor of poor clinical outcome in PM.

### 2.3 Driver mutations differentially regulate Hippo signalling in an isogenic, preclinical model of PM

**FIGURE 3:**
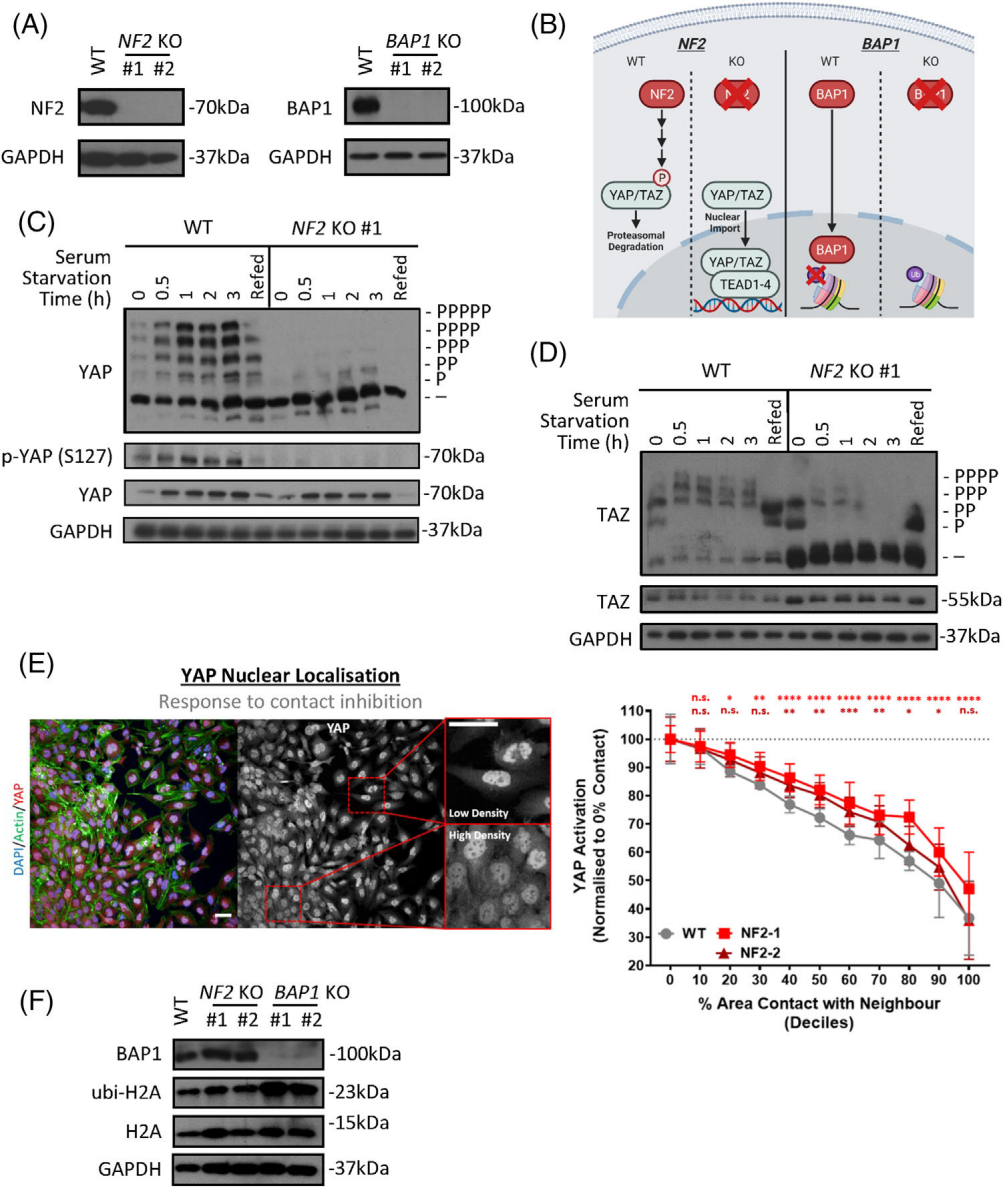
Development of an isogenic in vitro mesothelioma cell model of *NF2* and *BAP1* loss. (A) Western blots show successful CRISPR‐mediated knockout of *NF2* (left) and *BAP1* (right) in the mesothelial derived cell‐line, MeT‐5A. #1 and #2 label two independently generated knockout (KO) clones for each gene. (B) Schematic depicts well‐established functions of NF2 (left) and BAP1 (right) and therefore strategies of functional CRISPR KO validation, downstream of NF2 and BAP1. (C) Phos‐tag‐based western blots (top) show the phosphorylation status of YAP in response to serum starvation across a range of time points. Responses in wild‐type (WT) MeT‐5A cells (left) are compared to *NF2* KO #1 (right), with a clear loss of response evident in NF2 deficient cells. The same lysates as analysed by Phos‐tag are also developed on a regular Western blot (bottom), where the lysates are probed for levels of phosphor (S127)‐YAP, total YAP and GAPDH (loading control). Refed condition refers to cells starved of serum for 3 hours, before adding serum‐replete medium for 1 h. (D) Phos‐tag‐based western blots as in (C) but probed for TAZ, show phosphorylation status of TAZ in response to serum starvation, alongside total TAZ levels, in WT and *NF2* KO MeT‐5A cells. (E) Representative maximum projection images (left) obtained on the Operetta high‐content imaging platform show differential YAP nuclear compartmentalisation according to relative density in WT MeT‐5A cells. Scale bar = 50 μm. Scatter‐plot (right) shows the relative decrease in YAP nuclear localisation, as determined by the intensity of nuclear relative to cytoplasmic YAP, as percentage cell‐cell contact increases, with cells binned into deciles of percentage cell‐cell contact. *NF2* KO MeT‐5A cells (red) are less sensitive to fall‐off of YAP nuclear localisation as cells approach confluency than WT cells (grey), as determined by two‐way analysis of variance (ANOVA), adjusted for multiple comparisons. *n* = 8, with three wells acting as technical triplicates for each biological replicate. 300–9000 cells counted/well and a total of 76,901, 76,970, and 65,653 cells were quantified for WT, *NF2* KO clones #1 and #2 respectively. Error bars show SD from mean and significance levels illustrated above. n.s. = Not significant, *p < .05, **p < .01, ***p < .001 and ****p < .0001 relative to 0% contact with neighbour. (F) Western blot shows an increase in levels of (ubi)quityl‐histone H2A, an established BAP1 substrate, in *BAP1* KO cells, compared to WT and *NF2* KO clones

With YAP/TAZ activity established as a likely driver of PM progression, we sought to establish an isogenic model to probe Hippo pathway dysregulation in non‐malignant mesothelial cells in vitro. Two primary targets were selected for CRISPR‐Cas9 mediated knockout (KO) due to their high frequency of deletion in PM; *NF2*, to explore its role as a principal regulator of the Hippo pathway and *BAP1*, in order to assess its impact on YAP/TAZ activity, as well as more broadly, the effect of its loss on cancer progression, in the context of mesothelioma. Successful targeting of *NF2* and *BAP1* in the mesothelial cell line MeT‐5A is highlighted by the complete loss of protein in two independently established clones (Figure 3A). In order to characterise these isogenic KO clones, including that loss of either BAP1 or NF2 functionally recapitulates the expected biological impact within MeT‐5A cells, a range of downstream effects was assessed (Figure 3B).

NF2, a protein associated with the plasma membrane/cytoskeleton and which is known to temporally relocate to cell junctions,^55^, ^56^, ^57^ activates the Hippo kinase cascade in response to a range of stresses.^52^, ^58^ Firstly, serum starvation in multiple cell types, including HEK293A and U2OS cells, leads to LATS1/2 activation, and therefore YAP and TAZ phosphorylation and inactivation,^59^, ^60^ a phenomenon mediated by NF2.^61^ In the mesothelial context, a similar effect is observed, using Phos‐tag based western blots, a technique that allows for visualising YAP and TAZ phosphorylation status.^59^, ^62^ These Phos‐tag based gels reveal that *NF2* KO in MeT‐5A cells abrogates the serum starvation mediated YAP and TAZ phosphorylation readily observed in wild‐type (WT) cells (Figure 3C,D and Figure S3A). Of note, WT MeT‐5A cells exhibit a slight increase in levels of phosphorylated YAP/TAZ relative to *NF2* KO cells at steady state conditions; however, this effect is minor when compared to the difference seen upon serum starvation, with both WT MeT‐5A and independently generated NF2 KO clones containing very little detectable phosphorylated YAP/TAZ under serum‐replete conditions (Figure 3C and Figure S3A). Re‐expression of *NF2* in KO cells (Figure S3B) is sufficient to restore this serum starvation‐induced phosphorylation of YAP observed in WT cells (Figure S3C,D). We next used antibodies against YAP that are specific and verified for immunofluorescence‐based assays.^63^, ^64^ We used these on a confocal based high‐content imaging platform to quantify the nuclear localisation of YAP in MeT‐5A cells, to utilise as an activity marker for YAP. Surprisingly, this revealed that at steady state conditions, WT MeT‐5A display similar levels of nuclear YAP as compared to *NF2* KO cells (Figure S3E). Although unexpected, this is consistent with Phos‐tag‐based results (Figure 3C) showing high levels of active (unphosphorylated) YAP in WT cells at steady state, suggesting that NF2 may be inactive in these cells when cultured sparsely, with high levels of nutrients, and on plastic. Beyond mediating the activation of the Hippo pathway on serum deprivation, NF2 additionally orchestrates the response to contact inhibition,^65^, ^66^ which is the cellular response to cell‐cell contact and subsequent inhibition of growth. The use of a high‐content imaging system allows us to obtain large‐scale cellular datasets including percentage cell‐cell contact metrics for each cell, facilitating the analysis of nuclear, and thereby active YAP levels in response to contact inhibition. As cells approach confluency, WT MeT‐5A cells exhibit a robust decrease in nuclear:cytoplasmic ratios of YAP, indicating a reduction in activity, while *NF2* KO cells are markedly less sensitive to this effect (Figure 3E and Figure S3F). A decrease in nuclear YAP is, however, still observed in *NF2* KO MeT‐5A cells, consistent with previous findings showing additional NF2‐independent activation of the Hippo kinase cascade in response to contact inhibition.^61^

While BAP1 is reported to deubiquitinate a range of targets,^67^, ^68^, ^69^ it is most commonly associated with monoubiquitinated histone H2A (H2AK119Ub).^19^, ^70^ In order to validate the functional loss of BAP1 in MeT‐5A KO cells, we assessed levels of histone H2A ubiquitination at lysine residue 119. A robust increase in levels of ubiquitination of H2A is observed in *BAP1* KO cells, but not in *NF2* KO cells (Figure 3F). Recently, BAP1 was reported to deubiquitinate and thereby stabilise the LATS kinases in certain contexts.^48^ We, therefore, sought to establish if this effect is conserved in mesothelial cells. However, within mesothelial cells, BAP1 loss does not associate with a decrease in levels of LATS1/LATS2, as we conversely find that BAP1 loss drives an increase in both LATS1/LATS2 protein (Figure S3G). Despite being an inhibitory component of YAP/TAZ transcriptional activity, *LATS2*, which encodes the LATS2 kinase, is a direct target of YAP/TAZ and its expression is upregulated when the Hippo transcriptional module is active,^41^ driving a negative feedback loop within the Hippo pathway.^71^, ^72^ In order to assess whether LATS2 protein levels may be increased as a result of increased LATS2 expression due to YAP/TAZ activation, we quantified *LATS2* expression in *BAP1* KO relative to WT MeT‐5A cells, revealing a consistent, minor upregulation (Figure S3H). This suggests that cells may respond by activating the tumour‐suppressive Hippo kinase cascade as a negative feedback mechanism^72^, ^73^ in order to respond to a loss of the tumour suppressor BAP1.

### 2.4 BAP1 loss in vitro recapitulates general transcriptomic dysregulation observed in PM patients

**FIGURE 4:**
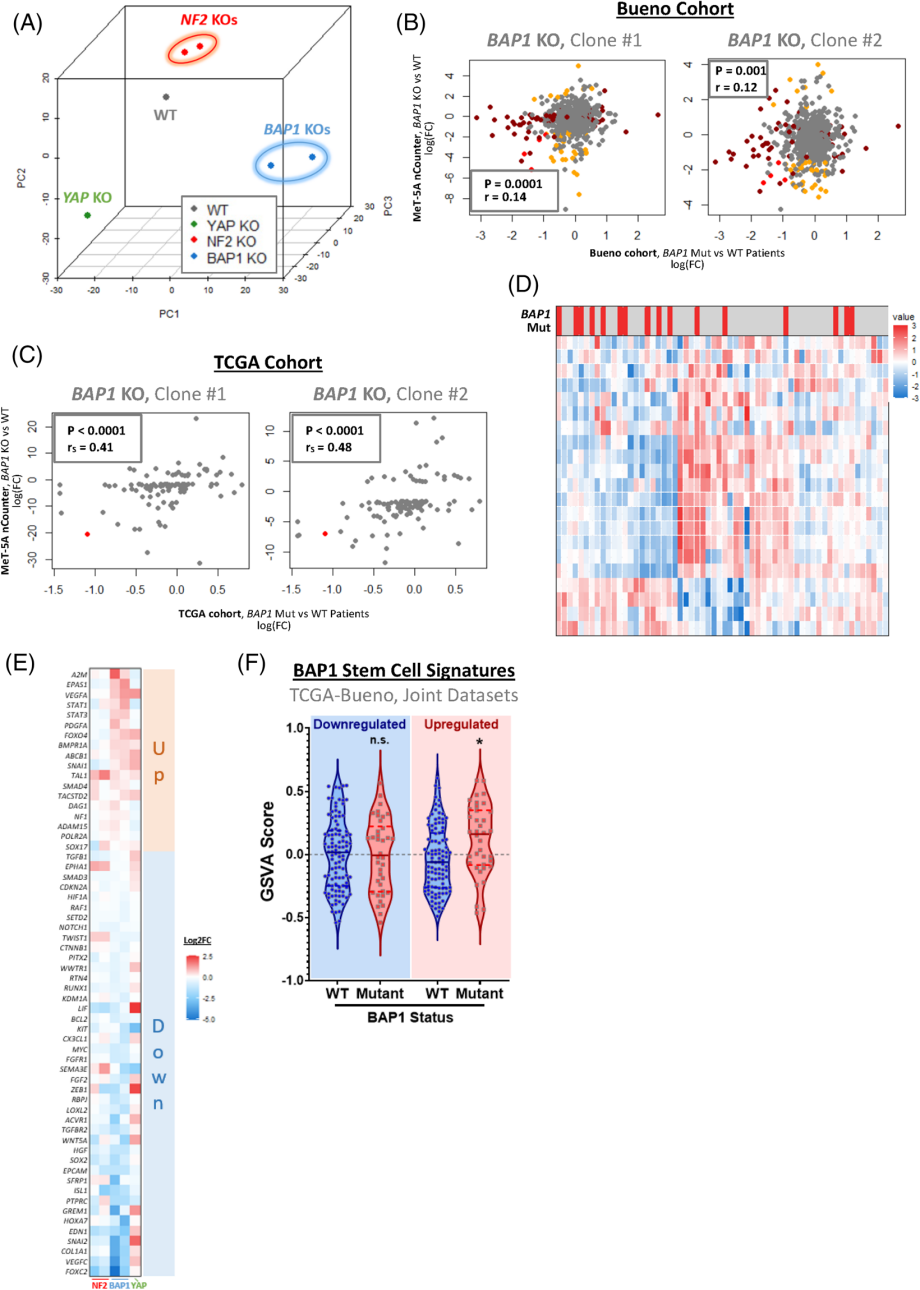
Gene dysregulation associated with pleural mesothelioma (PM) mutation status is preserved in in vitro models of *BAP1* loss. (A) 3D principal component analysis (PCA) plot shows MeT‐5A genotypes coloured according to knockout (KO) target and condition, with dimensionality reduction applied to the expression of 770 genes included in the NanoString PanCancer Progression nCounter panel. This highlights that transcriptional profiles diverge according to genotype and are consistent between paired KO clones. (B) Scatter plots show a correlation between fold changes in genes when comparing *BAP1* mutant versus wild‐type (WT) patients (x‐axis) and when comparing *BAP1* KO MeT‐5A cells to WT (y‐axis). Genes are coloured according to significance, with genes significantly differentially expressed in both clones of cell‐line models (orange), patients (dark red), and both (red) highlighted. There is a weak, though statistically significant positive correlation in fold‐changes calculated in patients compared to the in vitro model. (C) Scatter plots, as in (B), show correlation statistics of fold‐changes, comparing all genes quantified in the NanoString panel applied to MeT‐5A cell lines. In order to reduce noise, only genes identified to be reliably dysregulated in both KO clones in vitro are included, with genes significantly dysregulated in patient cohorts highlighted in red. A stronger positive correlation is observed when this additional complexity is removed. (D) Heat‐map shows the quantification of the expression of genes identified as significantly differentially expressed (|log(FC)| > 2.5 in both KO clones) in *BAP1* KO relative to WT MeT‐5A cells, as determined by NanoString, in patients from both cohorts grouped. The majority of those genes included are downregulated on *BAP1* KO in the in vitro model (with the exception of *IL18* and *NR4A3*), which is mirrored in *BAP1* mutant patients. Expression is shown in arbitrary units, with log(CPM) centred and scaled across each gene. As *BAP1* mutations are associated with a slight increase in YAP/TAZ activity, all patients with additional Hippo kinase module inactivating mutations (*NF2*, *LATS1*/*2*, *SAV1*) were excluded from the analysis. (E) Heatmap shows dysregulation of genes comprising stem cell maintenance, differentiation, and proliferation GO gene sets across MeT‐5A KO genotypes. Expression is shown as log2FC relative to WT MeT‐5A, with genes ordered according to the degree of dysregulation in *BAP1* KO cells. Up‐regulated (red) and down‐regulated (blue) gene sets were inferred according to the relative expression of *BAP1* loss. (F) Violin plot shows collective dysregulation of stem‐cell associated gene sets downregulated (left) and upregulated (right) in *BAP1* KO MeT‐5A cells within PM patients, as quantified via gene set variation analysis (GSVA). Patients combined from The Cancer Genome Atlas (TCGA) and Bueno datasets were grouped according to *BAP1* mutation status, with tumours exhibiting *BAP1* loss‐of‐function mutations (red) displaying a marked upregulation of the upregulated gene set as compared to WT *BAP1* samples (blue). As in (d) patients with Hippo kinase module inactivating mutations were excluded from the analysis. Correlation coefficients and *p‐*values in (B) and (C) were determined by Pearson and Spearman methods respectively, while *p‐*values in (F) were calculated via Dunnet's multiple comparison tests

With an in vitro model established and functionally validated at the molecular level, an important question remains: do these cell lines retain features intrinsic to patient tumours? In order to explore this, NanoString nCounter gene expression assays were conducted across MeT‐5A KO lines in order to quantify the expression of a panel of 1540 genes involved in cancer progression and immune signalling. Viewing expression broadly across the full panel reveals that separate clones from individual genotypes exhibit similar patterns of expression and cluster separately (Figure S4A). To further validate this observation, PCA demonstrates that variance in general transcription is driven by the various KOs, with clones of each KO exhibiting convergent transcriptional profiles (Figure 4A).

To compare the impact of the loss of tumour suppressors in vitro relative to in‐patient samples, we performed differential expression analysis to calculate fold‐changes in gene expression in *BAP1* WT vs *BAP1* mutant patients. These were then compared to fold‐changes calculated in WT vs *BAP1* KO MeT‐5A cells, with a significant, though relatively weak (mean *r* = 0.13), positive correlation in fold‐changes observed in both *BAP1* KO clones (Figure 4B). This highlights that the broad dysregulation of transcription affected by *BAP1* deletion in patients is generally preserved in *BAP1* KO MeT‐5A cells. In order to limit the noise inherent to transcriptomic analysis, with the majority of non‐differentially expressed genes potentially masking the correlation of genes truly disrupted upon *BAP1* loss, correlations were recalculated with genes limited to just those identified to be dysregulated within the cell‐line model. This reveals a relatively strong (*r* = 0.41 and 0.48 in KO clones #1 and #2, respectively) significant correlation between patient and cell‐line dysregulation (Figure 4C). Similarly, the clustering of patients according to an expression of genes identified to be highly dysregulated within *BAP1* KO cell lines shows a clear grouping of *BAP1* mutant patients (Figure S4B), which is even more pronounced when patients with Hippo kinase cascade inactivating mutations are excluded from analysis (Figure 4D). Additionally, splitting genes dysregulated in vitro into *BAP1* KO up‐ or down‐regulated gene sets and quantifying collective expression in patients categorised according to *NF2* and *BAP1* mutation status reveals that gene sets are selectively up‐or down‐regulated in *BAP1* mutant patients (Figure S4C), mirroring regulation in MeT‐5A cells. Taken together, these findings further validate that the impact of *BAP1* loss on widespread gene regulation in patients is retained within monocultured cells. This is evident despite the lack of an immune component or stroma, including tumour‐associated fibroblasts, suggesting that perturbation of expression is tumour cell‐intrinsic.

Animal models have shown that BAP1 is required for the switch from pluripotent to differentiated cells across a range of developmental lineages, including the mesoderm.^74^ In the context of cancers, such as uveal melanoma,^75^, ^76^, ^77^ BAP1 loss has been proposed as a driver of characteristics associated with cancer stem cells.^78^ To assess the role BAP1 depletion plays in mesothelial cells, we assessed the expression of genes associated with stem cell maintenance, differentiation, and proliferation, as determined via a search of gene ontology terms^79^, ^80^ across our NanoString data, within the generated *BAP1* KO MeT‐5A cells (Figure 3A). This revealed a selection of genes both weakly and strongly dysregulated in *BAP1* KO relative to WT cells (Figure 4E). Interestingly, this dysregulation appears to differentially affect homologues and functionally related genes: for example, *SNAI1*, which encodes the SNAIL transcription factor, a major effector of the stem‐like phenotype in multiple cancer types,^81^, ^82^ is found to be upregulated, while its homologue with shared functionality within cancer, *SNAI2* ^83^ (encoding SLUG) is downregulated on BAP1 loss (Figure 4E). While both act in combination to promote EMT and metastasis,^84^, ^85^ there is evidence that in certain contexts, each has a distinct role in driving cancer progression.^86^, ^87^ In patients with PM, expression of *SNAI1* and *SNAI2* is not correlated, as *SNAI1* is more frequently highly expressed within PM effusions.^88^ Similarly, *EPAS1*, which encodes the HIF‐2α transcription factor, a major regulator of the cellular hypoxic response, is upregulated on *BAP1* loss, while *HIF1A*, encoding HIF‐1α is downregulated. While both HIF transcription factors are known to contribute to tumorigenicity and cancer progression by orchestrating the tumour hypoxic response,^89^ HIF‐1α is generally considered a driver of metabolic reprogramming,^90^ with HIF‐2α coordinating a broader set of genes, including a variety of stem cell factors.^89^ This regulatory role of HIF‐2α is evidenced by the decrease in stem cell markers *NANOG* and *SOX2* and stem cell proliferation on *EPAS1* silencing in human embryonic stem cells.^91^ Interestingly, expression of YAP/TAZ signature genes, which are also known to potentiate EMT and cancer stem cell maintenance,^26^, ^53^, ^92^ are similarly dysregulated. Within MeT‐5A cells, eight of 22 genes comprising the signature are upregulated and seven downregulated in *BAP1* KO relative to WT cells (Figure S4D), with the remainder of target genes inconsistently dysregulated between *BAP1* KO clones.

Beyond this dysregulation, a variety of functionally similar genes associated with cancer progression were identified as upregulated in *BAP1* KO MeT‐5A cells. This includes *STAT1* and *STAT3*, which encode two STAT family transcription factors, both of which are involved in the maintenance of healthy and cancer stem cells,^93^, ^94^ while STAT3 additionally has been proposed as a target for treatment in PM.^95^ *PDGFA* and *VEGFA*, which both code for mitogenic growth factors known to stimulate mesenchymal proliferation associated with tumorigenesis are additionally upregulated in *BAP1* KO cells.^96^ To assess whether the dysregulation of stem cell‐associated genes observed in MeT‐5A cells is reflected in PM patients, we quantified and analysed the collective expression of both up‐ and down‐regulated gene sets in the joint TCGA and Bueno datasets. This revealed that the upregulated gene set is preserved within PM patients, with a collective expression of these genes also upregulated in patients with BAP1 mutations (Figure 4F). These observations suggest that this upregulated module of genes associated with stem cell maintenance may facilitate PM initiation by driving a stem‐like phenotype, as observed on BAP1 loss in uveal melanoma cancer cells and patients.^78^, ^97^

### 2.5 Exploring the transcriptional impact of NF2 and BAP1 loss in mesothelial cells

In order to infer the putative mechanisms of action by which NF2 and BAP1 loss regulates the observed transcriptional effect (Figure 4A), we assessed the top dysregulated genes within MeT‐5A KO cells, as determined by NanoString nCounter analysis. Within *NF2* KOs, this includes a variety of genes that, to our knowledge have not previously been directly associated with NF2, the Hippo pathway, or PM, including *KISS1*. *KISS1* is highly upregulated in MeT‐5A cells upon loss of NF2 and encodes the Kisspeptin family of G‐protein coupled receptor ligands. KISS1 has been reported to act as a context‐dependent inhibitor of metastasis or tumour promoter in a range of cancers,^98^, ^99^, ^100^ as well as a disruptor of proliferation and invasion in PM cell lines. Beyond upregulated targets, NF2 KO was identified to decrease the expression of *CXCL8*, which encodes interleukin‐8.

Expression of potential transcriptional regulated targets upon NF2 loss was validated via qPCR, which showed a close corroboration of dysregulation as those observed via NanoString (Figure S5A). Interestingly, there is a clear inverse correlation between directionality and the extent of dysregulation when comparing *NF2* KO MeT‐5A cells to *YAP* KO (Figure S5B), with genes upregulated on NF2 loss downregulated when YAP is lost and vice versa. This antagonistic relationship between NF2 and YAP, alongside NF2's established role as an upstream regulator of the Hippo pathway,^21^ suggests that YAP/TAZ transcriptional co‐activators and the TEAD family of transcription factors^30^, ^53^, ^101^ are likely effectors of *NF2* KO transcriptional dysregulation in our in vitro based MeT‐5A cellular PM model. To investigate this, we utilised the Cistrome Data Browser^102^ to visualise the binding of TEAD members to genes dysregulated on NF2 loss in cells across a wide range of widely‐used cancer cell lines, including MSTO‐211H, a cell line derived from PM tissue. This revealed peaks for TEAD1 and TEAD4 on both *KISS1* and *CXCL8* promotors (Figure S5C), suggesting that within cancer cells, TEAD transcription factors facilitate the expression of genes both up‐ and down‐regulated on NF2 loss. To facilitate the assessment of a broader selection of genes, we utilised Lisa (epigenetic Landscape In Silico deletion Analysis).^103^ Lisa is a tool developed to infer transcriptional regulators *in silico* likely to coordinate the regulation of gene sets, these analyses in Lisa combine publicly available ChIP‐seq data for specific regulators with markers of chromatin accessibility.^103^ Gene sets comprising potential transcriptional targets downstream of NF2 were generated by grouping together all genes identified as dysregulated on *NF2* KO (|log2FC| > 1). Both upregulated and downregulated genes were included, as it appears that transcription factors mediating the transcriptional dysregulation of NF2 loss do not coordinate unidirectional changes (Figure S5C). This analysis reveals the likely involvement of a number of transcription factors within the downstream transcriptional components of the Hippo pathway, as well as those known to associate with this component (Table 1). Most notably, this includes YAP and TEAD1, with YAP's regulatory role most significantly determined in the mesothelioma cell line, NCI‐H2052 (top two *p‐*values, each < .0001) and TEAD1 also yielding a highly significant association (*p* < .0001) in the MSTO mesothelioma line. Additional transcriptional regulators found significantly associated with gene‐set regulation include STAT3, which is known to bind to and regulate YAP/TAZ,^104^, ^105^ and FOSL2 and JUN, two components of the AP‐1 transcriptional complex, which co‐localises with YAP, TAZ, and TEAD at transcriptional enhancer sites.^53^, ^106^ Importantly, all three of these regulators recruit YAP/TAZ, orchestrating the transcription of YAP/TAZ target genes in transformed cells.^107^ Collectively, these findings further reinforce NF2 loss in the mesothelial context as a driver of YAP/TAZ‐TEAD mediated transcriptional perturbation.

**TABLE 1:** Lisa's analysis of NF2 knockout (KO) differentially expressed genes

**Transcription factor**	**Rank**	**1st Sample *p‐*value**	**2nd Sample *p‐*value**	**3rd Sample *p‐*value**	**4th Sample *p‐*value**	**5th Sample *p‐*value**
STAT3	1	5.22E‐16	1.48E‐05	2.11E‐05	1.05E‐03	5.80E‐03
SRPK2	2	5.15E‐08	5.59E‐01			
RELA	3	5.67E‐08	3.65E‐07	1.54E‐06	5.87E‐06	7.36E‐06
NR3C1	4	8.59E‐07	1.74E‐05	6.06E‐05	9.94E‐05	1.39E‐04
YAP1	5	9.23E‐07	2.46E‐06	5.44E‐04	2.34E‐03	8.45E‐03
FOSL2	6	1.84E‐06	5.92E‐05	1.36E‐04	1.87E‐04	2.47E‐04
SMAD3	7	3.02E‐06	1.10E‐04	1.57E‐04	2.04E‐04	2.81E‐04
JUN	8	3.94E‐06	2.42E‐04	2.46E‐04	1.16E‐03	2.47E‐03
TEAD1	9	5.21E‐06	2.19E‐05	3.08E‐05	8.05E‐05	1.62E‐01
TAL1	10	1.64E‐05	1.37E‐04	1.25E‐01	1.85E‐01	2.23E‐01

Results from Lisa's analysis,^103^ show the top 10 most significant *p‐*values across corresponding transcriptional regulators (including both transcription factors and chromatin regulators). The top five most significant samples, which consist of cell lines in which ChIP‐seq data corresponding to that regulator have been deposited in the Cistrome database, are included for each regulator.

### 2.6 NF2 loss in mesothelial cells cause YAP/TAZ hyperactivation in response to stress

**FIGURE 5:**
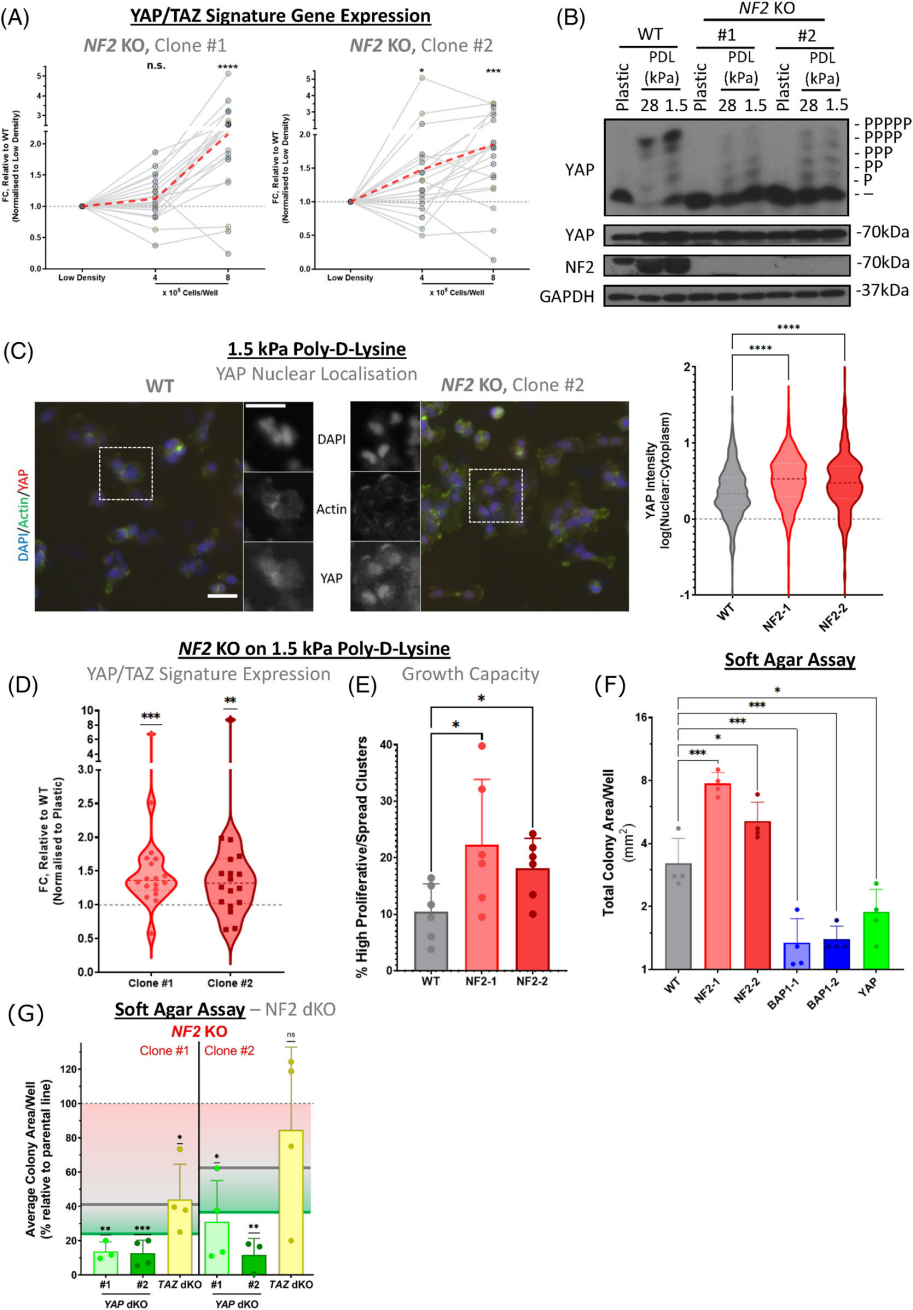
Culturing in conditions representative of the tumour microenvironment results in selective upregulation of YAP activity in cells deficient in NF2. (A) Line‐plot shows trends in expression of YAP/TAZ signature genes, as determined via quantitative polymerase chain reaction (qPCR), at increasing cell confluences. Each point represents the logFC of a single gene, relative to wild‐type (WT) expression at the same confluency and normalised to low density. The red‐dashed line shows the mean logFC calculated across the 22‐gene signature, with a clear increase in expression observed in both *NF2* knockout (KO) clones #1 (left) and #2 (right). logFCs were calculated by taking mean values across biological replicates (*n* = 5). (B) Phos‐tag‐based western blot shows the phosphorylation status of YAP in response to culturing MeT‐5A cells on a soft substrate. WT and *NF2* KO cells were seeded on poly‐D‐lysine coated plastic, 28 kPa, and 1.5 kPa ESS plates and left overnight before harvesting. WT MeT‐5A cells exhibit a clear induction of YAP phosphorylation (upshift) on both 28 and 1.5 kPa substrates, while the same response in NF2 deficient cells is markedly reduced. (C) Representative images (left), obtained via EVOS FL Auto 2 Imaging System, show dysregulation of YAP/TAZ activity, as determined by nuclear localisation, when culturing MeT‐5A cells on 1.5 kPa poly‐D‐lysine. Scale bar = 50 μm. A violin plot (right) shows quantification of YAP nuclear localisation, which is decreased on soft substrate relative to plastic (Figure S3E), with activity less diminished in *NF2* KO cells (>500 cells shown per genotype across four biological replicates). (D) Violin plot (left) shows the relative expression of YAP/TAZ signature genes, as determined via qPCR, in *NF2* KO cells, as compared to WT MeT‐5A (points represent mean expression per gene across three biological replicates). An increase in expression is observed, similar to the upregulation of YAP activity in the same condition (B). (E) Bar‐plot shows the percentage of highly proliferative/spread cell clusters on 1.5 kPa poly‐D‐lysine ESS plates. Clusters were determined as highly proliferative/spread if they exhibited areas > 10 times the median at time‐point 0 (*n* = 6). (F) Bar plots show quantification of the total area of colonies of MeT‐5A genotypes cultured in soft agar, with statistical significance relative to WT computed (*n* = 4). (G) Bar plots, as in (E), show the total area of colonies of dKO cells on an *NF2* KO background, cultured in soft agar. The area is normalised to corresponding parental *NF2* KO cells, with significance computed in comparison to parental genotype (*n* = 3–4). Annotations are included to highlight the mean relative total area of parental *NF2* KO cells (red line), WT MeT‐5A cells (grey line), and *YAP* KO cells (green line). *p‐*Values in (A), (C) and (D) were determined via a one‐sample Wilcoxon signed rank test, in (E) via Kruskal‐Wallis test, while *p‐*values in (F) were calculated via Dunnet's multiple comparison tests, with data log‐transformed to adjust for the skewness of soft agar assay results. Finally, *p‐*values for (G) were obtained using one sample t‐test, against a hypothetical mean of 100%. n.s. = Not significant, * *p* < .05, *** *p* < .001 and **** *p* < .0001 relative to low density (A, E, F), WT (C, D), or parental genotype (G)

While *in vitro BAP1* loss recapitulates the transcriptional disruption evident in *BAP1* mutant patient populations, the same phenomenon is not readily observed when compared with *NF2* KO cells (Figure S4E). This recapitulates that under steady‐state growth, with cells cultured in serum at relatively low density on plastic, NF2 does not appear to be highly active in WT MeT‐5A cells, with *NF2* loss having minimal impact on YAP/TAZ phosphorylation and nuclear localisation (Figure 3C and Figure S3E), while *NF2* KO cells display a transcriptional profile more similar to WT MeT‐5A cells relative to other genotypes (Figure 4A and Figure S4A). In order to assess a relevant metric of gene dysregulation on *NF2* loss, expression of YAP/TAZ signature genes, which are consistently upregulated in patients with Hippo kinase module inactivating mutations (Figure 2B), was quantified in *NF2* KO cells. To further contextualise this given NF2's confirmed role as a regulator of Hippo signalling in response to stresses associated with the tumour microenvironment, including serum starvation (Figure 3C) and contact inhibition (Figure 3E), expression was assessed at increasing cell densities. This analysis reveals that, as *NF2* KO cells become more confluent, they exhibit a consistent general upregulation of signature genes^41^ relative to WT MeT‐5A cells, with a mean increase in expression of the entire signature of 85% and 115% across two *NF2* KO clones compared to WT cells (Figure 5A). These data closely resemble those from *NF2* mutant patients (Figure 2B), indicating that Hippo kinase cascade activation in response to contact inhibition is impaired on *NF2* loss in vitro.

The stiffness of cell culture plastics is well in excess of 100 kPa, approximating 10^6^ kPa,^108^, ^109^ which is much stiffer than tissue. To more faithfully in vitro recapitulate the environment at the onset of PM, cells were next cultured on poly‐D‐lysine with an elasticity index of 1.5 kPa, which matches the mechanical properties of pleural tissue.^110^, ^111^ Phos‐tag based western‐blots revealed that YAP phosphorylation is increased when cells are cultured on soft poly‐D‐lysine, with a clear shift particularly observed when comparing WT MeT‐5A cells cultured on 1.5 and 28 kPa substrate, relative to cells cultured on plastic (Figure 5B). However, this effect was considerably less pronounced in cells lacking NF2, with only a minor increase in YAP phosphorylation (Figure 5B). Similarly, while YAP nuclear localisation is also generally decreased on soft substrate, *NF2* KO is less affected than WT MeT‐5A cells (Figure 5C and Figure S3E). Our quantification highlights a significant increase in nuclear YAP signal in *NF2* KO relative to WT cells (Figure 5C), similar to when culturing at high cell density (Figure 3E). A concomitant relative upregulation of the YAP/TAZ signature is also observed in *NF2* KO cells (Figure 5D), further validating the activation of YAP/TAZ reflected in the dysregulation of the signature in *NF2* mutant patients (Figure 2B). Beyond this, after 48 h growth on a soft 1.5 kPa substrate, ∼20% of *NF2* KO cell clusters reached >10 times the median size at time point 0, as compared to 10% of WT cells (Figure 5E). This higher percentage of large colonies upon NF2 loss indicates either a greater proliferative capacity on a soft substrate or a resistance to the expected decrease in the cytoplasmic area observed when cells are cultured on surfaces with low elastic moduli.^112^, ^113^ Moreover, *NF2* KO cells exhibit greater anchorage‐independent growth, as determined by soft agar assays (Figure 5F), which combined indicate a greater potential for tumorigenicity on *NF2* loss. For these functional assays, cells are cultured in 0.35% soft agar, which is reported as having Young's modulus around 30–50 kPa,^114^, ^115^ a stiffness where we observe a clear increase in active YAP in *NF2* KO compared to WT cells (Figure 5B). In order to establish if the phenotypes observed in *NF2* KO cells are mediated by, and dependent on, YAP or TAZ, we established *NF2*‐*YAP* double knockout (dKO) and *NF2*‐*TAZ* dKO MeT‐5A cells (Figure S3J). The capacity to readily grow in soft agar is markedly decreased in *YAP* KO, as well as in *NF2*‐*YAP* dKO) cells (Figure 5F,G). While *NF2*‐*YAP* dKO is sufficient to inhibit colony growth in soft agar, *NF2*‐*TAZ* dKO has a modest impact, with total area coverage roughly equivalent to WT MeT‐5A cells (Figure 5G). These observations suggest that *NF2* KO MeT‐5A cells’ capacity for anchorage‐independent growth is primarily driven by hyperactive YAP, with cells lacking NF2 exhibiting dependence on YAP for this trait. This indicates transcriptional addiction, a common feature of tumour cells.^116^, ^117^ Despite YAP's primary role in facilitating anchorage‐independent growth, we anticipate that targeting both YAP and TAZ might be more effective and may be necessary in a clinical setting.^53^ Interestingly, *BAP1* loss decreases anchorage‐independent growth (Figure 5F), with decreased total colony coverage on soft agar, even as compared to *YAP* KO MeT‐5A cells. This might reveal that cellular BAP1 loss is only advantageous in settings where the cancer cells are under additional stresses,^118^ such as upon nutrient limitations in the cancer niche and the onset of the Warburg effect.^118^

Although transcriptional dysregulation via YAP/TAZ is observed on *NF2* KO in cells cultured at low cell density on plastic (Figure S5A–C), the addition of stresses, some of which were previously associated with NF2 activation, drives a greater degree of relative YAP/TAZ activation and subsequent transcriptional effects. These findings, therefore, highlight the role NF2 plays as a facilitator of the Hippo pathway's mechanosensory component,^53^, ^119^ acting to inhibit YAP/TAZ activity in response to serum starvation (Figure 3C,D), cell density (Figure 3E), and decreasing substrate stiffness (Figure 5B–D), with NF2 loss rendering cells less sensitive to growth on soft substrate. Within the scope of PM, the increase in context‐dependent YAP/TAZ activation on NF2 loss likely translates to a decreased sensitivity to and enhanced proliferative potential within, a tumour niche, with a concurrently increased capacity for anchorage‐independent growth.

## 3 DISCUSSION

The Hippo signalling pathway is closely tied to a range of oncogenic pathways across a wide variety of cancer types^51^ and is found frequently, mutationally perturbed in PM relative to other cancers.^58^ Although incidence rates of PM are relatively low and there is, therefore, a limited selection of well‐annotated, high‐throughput clinical datasets available to analyse, we here show with confidence, via pooling the largest PM datasets available,^12^, ^13^ that Hippo signalling disruption is closely associated with worse prognosis in PM patients. The inclusion of the larger Bueno cohort^13^ as part of these analyses facilitates enhancing the statistical power of included tests, increasing confidence while expanding on recent work which assessed the clinical impact of Hippo dysregulation within the numerically smaller TCGA cohort.^40^, ^41^, ^120^ These findings are also consistent with a recent study showing that *NF2* deletion tends to be positively selected for during tumour evolution and might be a late‐stage event,^121^ all suggesting that activation of the Hippo transcriptional module may be a limiting factor for PM progression and metastasis. Our preclinical modelling of *NF2* loss supports this observation, with in vitro *NF2* knockout in mesothelial cells facilitating resistance to YAP/TAZ inactivation in response to serum starvation, contact inhibition, and culturing on soft substrate (Figures 3C–E and 5A–C). This is of relevance in a clinical setting as nutrient deprivation is a recurring feature of solid tumours as a result of poor vascularisation and high energy demand,^122^ while tumour tissues in PM are typified by hypoxia, suggesting limited vascularisation and hence nutrients.^123^, ^124^ Loss of contact inhibition is a critical and primary hallmark of cancer cells,^125^ while tumour growth, infiltration and metastasis are all limited by contact inhibition of proliferation.^126^, ^127^ Additionally, the establishment of the tumour microenvironment and metastatic niche over time is associated with extracellular matrix remodelling and a concurrent change in stiffness.^128^, ^129^ Our data point to NF2 being a cardinal sensor of mechanotransductive processes in mesothelial cells, and that the loss of NF2 therefore might play an integral part in mesothelioma during disease progress. This possibility is reinforced by the YAP addiction observed in *NF2* KO cells when assessing anchorage‐independent growth in vitro (Figure 5G). These findings collectively point to a function of Hippo pathway kinase inactivation to promote tumour growth under conditions of stress commonly experienced within tumour tissue, with YAP and TAZ known to generally enhance cell growth and survival under nutrient‐limited conditions,^63^, ^130^ resistance to therapeutics^53^ and motility in response to cytoskeletal tension.^131^

Considering the importance of perturbed Hippo signalling in driving PM, therapeutic interventions that inhibit the downstream effectors of the pathway are likely to be instrumental in future treatment regimens.^53^ Previous studies have revealed potential in inhibiting the individual protein products of various YAP/TAZ signature genes in diverse cancer types,^132^, ^133^ an example of such is bemcentinib, an inhibitor of AXL whose expression is directly mediated by the Hippo transcriptional module, which has been combined with pembrolizumab in recent clinical trials in PM.^134^ However, as the Hippo pathway regulates the expression of a wide variety of genes associated with oncogenic pathways, a likely more productive approach may be to directly target the transcriptional effectors (YAP, TAZ and the TEAD transcription factors).^53^ Efforts to target this transcriptional module identified the benzoporphyrin derivative verteporfin as a disruptor of the YAP‐TEAD complex with anti‐cancer potential.^135^, ^136^ However the drug is effective at high concentrations and appear to lack specificity^137^ limiting its clinical utility in cancers. In recent years, there has been a major push to identify viable drugs that directly target the transcriptional module of the Hippo pathway and can be used in the clinic, either by disrupting the interaction between YAP/TAZ and TEAD,^138^, ^139^ or by targeting TEAD stability.^140^ While the various novel therapeutics identified in this fashion have yet to be fully validated in a clinical setting, early findings are promising,^53^ and with some already in early‐stage clinical trials. The wide interest in targeting YAP/TAZ‐TEAD in cancers,^53^ and the range of currently developed compounds targeting this transcriptional modality highlight the need for relevant preclinical models. The ability to evaluate these compounds effectively in order to achieve their full therapeutic potential in the near future includes the further development and assessment of their mechanism of action. These translational efforts rely on the development of scalable and clinically relevant model systems, such as the one developed here.

Although we present evidence that BAP1 loss in certain instances may be involved in partially activating the Hippo transcriptional module (Figures S2B and S4D), the mechanism by which this is effected in vitro in mesothelial cells is unclear, though not mediated via degradation of LATS proteins as previously described within the pancreatic cancer context^48^ (Figure S3G,H). A core role for BAP1 is to act as a deubiquitinase as part of the PR‐DUB complex^20^ and BAP1 has been described as a regulator of a variety of epigenetic markers.^118^ This, therefore, suggests that BAP1's involvement in regulating the Hippo pathway is likely indirect, possibly as a result of the transcriptional changes effected by epigenomic alterations on *BAP1* loss; this is all the more relevant as the primary metric with which YAP/TAZ activity is quantified clinically in this study is transcriptional in nature (Figures 1, 2 and 5A–D). Additionally, BAP1's ability to modulate expression broadly via Polycomb repressive complexes^20^ may explain the faithful recapitulation of patient dysregulation in vitro, in the absence of non‐tumour cell interactions. This observation is particularly relevant as these epigenetic writers tend to exhibit context sensitivity.^141^

Interestingly, we find that BAP1 loss in vitro leads to a reduction in invasive potential. This finding is consistent with previous studies, which show that, while ectopic BAP1 expression may lead to enhanced anchorage‐independent growth in renal cancer cells,^142^ BAP1 depletion via KO or KD inhibits proliferation, colony formation, and anchorage‐independence in cells from multiple cancer types.^67^, ^78^, ^143^ Mechanistically, BAP1 loss has been proposed to act by driving dedifferentiation and stem‐like characteristics associated with early cancer rather than tumorigenicity.^78^ Within the mesothelial context, we find that BAP1 loss leads to the dysregulation of a range of genes involved in stem cell specification, with a module of these genes consistently upregulated within both the generated isogenic cell‐line model and tumour tissue (Figure 4E,F). Additional work is required to determine how this dysregulation drives dedifferentiation within mesothelial cells and how this may affect PM progression. However, the decrease in invasiveness observed on *BAP1* KO in vitro may in part explain the observation in other studies that BAP1 loss predicts improved relative prognosis in PM cases.^45^, ^144^ Although the same effect is not apparent in the larger, combined cohorts (Figure S1E), this may be limited by inferring BAP1 loss by mutation status alone. Taken together, this highlights BAP1's complex role as a tumour suppressor,^118^ whereby its high frequency of loss strongly implicates its involvement in driving PM onset, while in the mesothelial context inhibits anchorage‐independent growth (Figure 5F) and sensitises tumour cells to therapeutic intervention.^45^

In conclusion, this work serves to reinforce the role that Hippo pathway dysregulation plays in driving PM progression, which is increasingly relevant as inhibitors of the transcriptional module of the pathway are likely to become clinically available.^53^ We also highlight the potential in stratifying PM patients according to biomarkers of YAP/TAZ activity rather than driver mutation status, while developing, establishing, and validating a robust isogenic preclinical model. This isogenic cellular PM model is a unique system and provides a platform to allow for further discoveries and in‐depth insights into harnessing the therapeutic potential of loss of BAP1 and NF2 in mesothelioma, as well as provide a detailed understanding of BAP1 and the Hippo pathway's fundamental biological functions. Currently, the cellular model system has not yet been analysed in vivo. Noteworthy, a range of gene‐sets, including cytokines as well as ECM components, are widely dysregulated (Figure 4A). Xenografts using the developed model system may shed further light on the interplay between PM driver mutations and the onset/progression of PM, including revealing critical insights into the interplay between the cancer cells and components of the stroma including the immune system. Consequently, we envision that the isogenic preclinical cellular model developed and characterised here is likely to be impactful in the context of developing future stratified approaches in PM.

## 4 MATERIALS AND METHODS

### 4.1 Culture maintenance

MeT‐5A mesothelial cells were cultured in RPMI‐1640 (31870‐025; Gibco) supplemented with 10 mM HEPES Buffer solution (15630‐049; Gibco), 2 mM L‐Glutamine (25030‐024; Gibco), 10% foetal bovine serum (FBS) (10500‐064; Gibco), with 100 units/ml of penicillin and 100 μg/ml of streptomycin (15140‐122; Gibco). HEK293T cells were kindly gifted by Professor Kun‐Liang Guan (UCSD) and cultured in high glucose Dulbecco's Modified Eagle Medium (21969‐035; Gibco) supplemented with 2 mM L‐Glutamine (25030‐024; Gibco), 10% FBS (10500‐064; Gibco). All cells were cultured in the presence of 100 units/ml of penicillin and 100 μg/ml of streptomycin (15140‐122; Gibco) and incubated at 37°C with 20% O_2_. For modelling soft tissue substrate, cells were cultured on 100 μg/ml poly‐D‐lysine (P7886; Sigma‐Aldrich), chosen due to its inertness with regards to the Hippo pathway.^64^ Poly‐D‐lysine was coated on 1.5 and 28 kPa Ibidi ESS plates (81291; Ibidi) and cells were cultured overnight before imaging and protein‐lysate/RNA harvesting. Cells were subject to routine checks for mycoplasma using a MycoAlert Mycoplasma Detection Kit (LT07‐318; Lonza).

### 4.2 Gene knockout and re‐expression

CRISPR‐Cas9 mediated KO was carried out using the following guide RNA (gRNA): 5′‐GTCCATGGTGACGATCCTCA‐3′ for *NF2* #1; 5′‐GAGTTCAATTGCGAGGTAAC‐3′ for *NF2* #2; 5′‐AAACGGACCGGCGCTCTTCGATCC‐3′ for *BAP1* #1; 5′‐CACCGGATCGAAGAGCGCCGGTCC‐3′ for *BAP1* #2; 5′‐CACCGCATCAGATCGTGCACGTCCG‐3′ for *YAP1* (YAP); and 5′‐ CACCGTGTCTAGGTCCTGCGTGACG‐3′ for *WWTR1* (TAZ). gRNA sequences were ligated to the pSpCas9(BB)‐2A‐Puro CRISPR construct (PX459; Addgene, #48139) as described in Rausch et al.^62^ MeT‐5A cells were electroporated for 20 ms at 1400 V, pulsed once using the Neon electroporation transfection system (MPK10096; Invitrogen). After overnight recovery, the cells were selected for 48 h with puromycin to enrich for cells that had taken up the plasmid. Cells were then taken out of selection for 24 h before fluorescence‐activated cell sorting (FACS) into single wells of a 96‐well plate. Clonal expansion and replicate plating were carried out after FACS‐based single‐cell sorting. KO validation was performed by western blotting. *NF2* re‐expression in *NF2* KO MeT‐5A cells was achieved via lentiviral transduction using a pBABE puro system. Lentivirus was produced in HEK293T cells and harvested at 48 and 72 h post‐transfection, where the supernatant is filtered with low binding 0.45 μm SFCA filters (Corning, 431220), and added to polybrene supplemented MeT‐5A cells directly or stored at ‐80°C for later use. Selection after transduction/transfection was achieved with puromycin treatment.

### 4.3 Western blotting

Cell lysates were harvested and analysed using home‐cast sodium dodecyl sulfate (SDS) polyacrylamide gels to perform western blots as in Hansen *et al*.^63^ PageRuler prestained Protein Ladder (26616; Thermo Scientific) was used to provide a scale for protein size and separate proteins transferred from gels to Immobilon‐P PVDF membranes (IPVH00010; Millipore). After transfer, the membrane was blocked in 5% milk in tris‐buffered saline‐T (TBS‐T) (150 mM NaCl, 20 mM Tris, 0.1% TWEEN 20), washed with TBS‐T, and incubated with primary antibodies diluted in TBS‐T supplemented with 2% bovine serum albumin (Fisher Bioreagents). Membranes were then washed and incubated for 1 h with secondary antibodies specific to the primary antibody isotype, conjugated to Horseradish Peroxidase (HRP; P044801‐2 and P044701‐2, Agilent). Finally, Immobilon Western Chemiluminescent HRP Substrate (WBKLS0500; Millipore) was used to produce a visible signal from HRP, which was subsequently developed using X‐ray film (MOL7016; SLS). Phos‐tag gels were generated by adding Phos‐tag reagent (304‐93521; Alpha Laboratories) and 10 mM MnCl_2_ added to each SDS polyacrylamide gel. Primary antibodies used were as follows: NF2 (D1D8; CST), BAP1 (13271S; CST), YAP (ab52771; Abcam), phospho‐YAP (Ser127; 13008, CST), histone H2A (12349; CST), ubiquityl‐histone H2A (Lys119; 8240, CST), LATS1 (3477; CST), LATS2 (3477; CST), and GAPDH (SC‐47724; Santa Cruz) used as a loading control for samples.

### 4.4 Quantitative polymerase chain reaction and NanoString nCounter

Cells were seeded in a 6‐well plate and allowed to adhere overnight. RNA was harvested from plates and purified using the RNeasy Mini Kit (Qiagen) according to the manufacturer's specifications. Purified RNA was quantified via NanoDrop spectrophotometer and kept frozen at ‐80°C until needed. RNA was submitted to the University of Edinburgh's Host and Tumour Profiling Unit (HTPU) for nCounter analysis with PanCancer Progression and Immune Profiling panels used, conducted according to the manufacturer's specifications. Each panel consisted of 770 genes, with an overlap of 157 gene targets between panels; 719 and 486 genes were detected in any sample in the Progression and Immune Profiling panels, respectively. For quantitative polymerase chain reaction (qPCR), cDNA was generated using 100 ng of RNA as input with a High‐Capacity cDNA Reverse Transcription Kit (4368814; Applied Biosystems), with the reaction carried out according to all manufacturer's instructions. cDNA was frozen at ‐20°C until required for qPCR, with assays carried out using Brilliant III Ultra‐Fast SYBR Green QPCR Master Mix (600883; Agilent) and custom IDT primers, designed using templates deposited on PrimerBank^145^ all according to manufacturer's directions. Assays were performed on a QuantStudio 5 Real‐Time PCR System, with data processed and analysed using R statistical software. Primer sequences were as follows: 5′‐GCTCGTTGAGTGAACGGCT‐3′ and 5′‐CATGAGCTAGTACAACATGAGGG‐3′ for *AMOTL2*; 5′‐AGTAGAGGAACTGGTCACTGG‐3′ and 5′‐TGTTTCTCGCTTTTCCACTGTT‐3′ for *ANKRD1*; 5′‐CCAAGGTGAGCTTTCCCTCG‐3′ and 5′‐CCTACTAGACCATAGGTCGTCGT‐3′ for *ARHGEF17*; 5′‐TAGAACAGCCCTTCAGAAAGTGA‐3′ and 5′‐CGGGGTTGTCTCGACTTAAAAA‐3′ for *ASAP1*; 5′‐GTGGGCAACCCAGGGAATATC‐3′ and 5′‐GTACTGTCCCGTGTCGGAAAG‐3′ for *AXL*; 5′‐CCCTGTGACGAGTCCAAGTG‐3′ and 5′‐GGTTCCGTAAATCCCGAAGGT‐3′ for *CRIM1*; 5′‐GAGGCAGAAGTACGGGGTTG‐3′ and 5′‐CAGGAATCACGGTTTCATGCT 3′ for *DOCK5*; 5′‐GGCGCTTCAGGCACTACAA‐3′ and 5′‐TTGATTGACGGGTTTGGGTTC‐3′ for *F3*; 5′‐GCTGGTGGACCTAGTACAATGG‐3′ and 5′‐CTTACGAGCCGGTCGAAGTTG‐3′ for *FJX1*; 5′‐AATGCCACTCGCCCTACAC‐3′ and 5′‐CGTTCTGGTGCAAGTAGCTCT‐3′ for *FOXF2*; 5′‐GAGAGCAGAAGACCGAAAGGA‐3′ and 5′‐CACAACACCACGTTATCGGG‐3′ for *GADD45A*; 5′‐AGAGCACAGATACCCAGAACT‐3′ and 5′‐GGTGATTCAGTGTGTCTTCCATT‐3′ for *IGFBP3*; 5′‐ACTTTTCCTGCCACGACTTATTC‐3′ and 5′‐GATGGCTGTTTTAACCCCTCA‐3′ for *LATS2*; 5′‐TAATTGGCACGGCGACTGTAG‐3′ and 5′‐GGAGATCAGCTTGTACGGCAG‐3′ for *MYOF*; 5′‐GCCTGGGAGCTTACGATTTTG‐3′ and 5′‐TAGTGCCCTGGTACTGGTCG‐3′ for *NT5E*; 5′‐CGCCCAAGCCCCTAATGAAG‐3′ and 5′‐TCCCTCCGTATGTGCATCAGA‐3′ for *NUAK2*; 5′‐ATGCCTTTTGGTCTGAAGCTC‐3′ and 5′‐CCCTGTGCTTTCCACCGAC‐3′ for *PTPN14*; 5′‐GGGGAACAGTTGAGTAAAACCA‐3′ and 5′‐ACAATTTTTCCATACGGTTGGCA‐3′ for *RBMS3*; and 5′‐CAGCACACTCGATATGGACCA‐3′ and 5′‐CCTCGGGCTCAGGATAGTCT‐3′ for *TGFB2*. All gene expression values were normalized to *Hypoxanthine Phosphoribosyltransferase 1* (*HPRT1*) expression, with primer sequence 5′‐AGAATGTCTTGATTGTGGAAGA‐3′ and 5′‐ACCTTGACCATCTTTGGATTA‐3′.

### 4.5 Immunofluorescence

For steady‐state imaging, cells were seeded on 96‐well μClear plates (655090; Grenier). For mixed models, *NF2* KO cells were stained with CellTracker Red CMTPX Dye (C34552; Invitrogen) for 30 min before seeding. Cells were fixed using 4% formaldehyde (28906; Thermo Scientific), rinsed with PBS and then permeabilized in IF buffer (2.5% FBS and 0.3% Triton X‐100 in PBS). Fixed, permeabilised cells were next incubated overnight with a specific YAP antibody^64^ (ab52771; Abcam), before multiple PBS washes and further incubation with DAPI, phalloidin conjugated to Alexa Fluor 488 (A12379; Invitrogen) and Alexa Fluor 555/647‐conjugated goat anti‐rabbit secondary antibody, (A‐21428 and A27040; Invitrogen). Fluorescence was then imaged using the Operetta or Opera Phenix Plus high‐content imaging systems (PerkinElmer) using a 20x objective for 96‐well plates. Eight biological replicates were conducted with cells seeded at 7500 cells per well and imaged at a range of time‐points. 25 fields were imaged per well, with three wells per sample/condition to act as technical controls. At the time of imaging, wells contained between 300 and 9000 cells. Cell features were computed using the Columbus image data storage and analysis system (PerkinElmer) and statistical analysis was then carried out using R and plotted with GraphPad Prism. Variance across samples was adjusted by removing outlying biological replicates, with outliers specified as values greater than 2 median absolute deviations from the median of biological replicates. Ibidi ESS plates were imaged using the EVOS FL Auto 2 Imaging System (Invitrogen) at 10x magnification, with individual cells sampled from the total population to allow for identical numbers across four biological replicates for statistical testing.

### 4.6 Data acquisition and processing

TCGA‐MESO data were accessed and downloaded using the GDCquery function, while TCGA RPPA data were obtained using the GDAC Firehose repository (https://gdac.broadinstitute.org/). Raw data from the Bueno cohort were obtained using the pyEGA3 download client and aligned using the STAR aligner^146^ with default settings. HTSeq^147^ was used to generate count tables post alignment, with normalisation via trimmed mean of M‐values implemented via the edgeR package.^148^ All data were then imported into the R environment for additional analyses, with pooled data obtained by combining both TCGA^12^ and Bueno et al.^13^ cohorts and normalising with the ComBat function within the SVA package.^149^ Differential expression analysis was conducted on un‐normalised count data using DESeq2.^150^

### 4.7 Stiffness and soft agar assays

The growth of cells on poly‐D‐lysine coated ESS plates was assessed by seeding 500 cells into cloning rings (8 mm × 8 mm; C1059‐1EA, SLS). Cells were fixed 24 or 72 hours post‐seeding (representing 0‐ and 48‐h time‐points, respectively) and stained with phalloidin conjugated to Alexa Fluor 488, in order to infer cell coverage. Plates were imaged at 10x magnification with the EVOS FL Auto 2 Imaging System, with cell cluster boundaries and corresponding cluster areas then computed using CellProfiler. For soft agar assays, MeT‐5A cells were split and resuspended in 0.35% agar dissolved in 2X strength MeT‐5A culture medium, prepared using powdered RPMI‐1640 (51800‐019; Gibco). Cell suspensions in 2 ml agar were then seeded onto 6‐well plates coated with 1% agar, with 5,000 cells per well. After one week of growth, cells were fixed and stained using a mixture of crystal violet (0.5%, 11435027; Thermo Scientific) and methanol. Bright‐field images of plates were then captured using the EVOS FL Auto 2 Imaging System at 10x magnification, with ImageJ used to quantify the total colony area/well. For both ESS plate and soft agar assays, image capture was automated, with 35%–50% of wells (300–500 fields/well total) imaged and stitched together.

## CONFLICT OF INTEREST

The authors declare they have no conflict of interest.

## Supporting information


**Supplementary Figure 1| Broad mutation profiles are insufficient to define patient populations with distinct clinical outcomes**.
**a,** PCA plots displayed as in figure 1d, show PCs along different axes to highlight clusters. **b,** PCA plots, depicted in 3D, show patients coloured according to mutation status. Broad transcriptional profiling is insufficient to distinguish between the populations of patients categorised by mutation of *BAP1* and *NF2* in the TCGA (left; n = 86) and Bueno *et al* (right; 98) cohorts. **c,** Barplot shows the percentage of patients classified by consensus subtypes as generated in Bueno *et al* ^16^, with patients split according to mutation status of *BAP1* and *NF2*. n = 98. **d,** Barplot, as in (c), highlights the absence of significant association between mutation status and T‐staging across the merged TCGA and Bueno *et al* datasets (n = 163). **e,** Kaplan‐Meier curves show overall survival of patients split according to mutation status, with patients categorised as exhibiting either *NF2* or *BAP1* mutations, as well as mutations in both or neither. No significant difference in survival across the patient populations is observed. *P* values for (c) and (d) determined via Fisher's exact test, while *P* values for (e) were calculated via log‐rank test.

**Supplementary Figure 2| Analysing TCGA and Bueno cohorts separately highlights consistency between datasets**.
**a,** Violin plots show GSVA scores of YAP/TAZ signature gene expression as in figure 2a, with results split by dataset. A significant collective overexpression of this gene‐set is observed in patients with Hippo kinase cascade inactivating mutations in both TCGA (left; n = 86) and Bueno *et al* (right; n = 98) cohorts. **b,** Violin plots as in (a), show signature scores in patients split according to *BAP1* mutation status, excluding patients harbouring Hippo pathway inactivating mutations. While there is an association between *BAP1* mutation and expression of YAP/TAZ target genes, this is minor and only seen to be significant in the TCGA (left) and not Bueno *et al* (right) cohort. **c,** Kaplan‐Meier curves show overall survival of patients split according to YAP/TAZ signature thresholds. Patients classed as YAP/TAZ signature display a pronounced reduction in overall survival in both TCGA (top; HR = 4.4, n = 86, threshold at 74%) and Bueno *et al* (top; HR = 1.76, n = 211, threshold set at 52%) cohorts. **d,** Bar‐plots, as in figure 2e, show tumour stage in patients categorised as signature high/low. *P* values in (a) and (b) were determined by Mann‐Whitney U test, *P* values and hazard ratios for (c) were calculated via log‐rank test and Cox proportional hazard model respectively, while *P* values for (d) were calculated via Fisher's exact test. n.s. = Not significant, *P < 0.05, **P < 0.01, and ****P < 0.0001 relative to WT.

**Supplementary Figure 3| Validation of *NF2* KO mediated activation of YAP**.
**a,** Phos‐tag based western blots (top), as in as in figure 3c, shows phosphorylation status of YAP (left) and TAZ (right) in response to serum starvation across a range of time‐points. Responses in WT MeT‐5A cells are compared to the second *NF2* KO clone (#2), with a similar decreased sensitivity to starvation observed on NF2 loss in *NF2* KO #1. A standard SDS‐gel based Western blot (bottom) is also shown with the same samples analysed, highlighting levels of phospho‐YAP (S127) and YAP (left), TAZ (right), together with GAPDH. **b,** Western blots showing NF2 expression levels in WT, *NF2* KO, and re‐expression of *NF2* in *NF2* KO MeT‐5A clones. **c‐d,** Phos‐tag based western blots, as in (a), with *NF2* re‐expressed in *NF2* KO MeT‐5As. Response to serum starvation is restored upon *NF2* re‐expression, with increased phosphorylation of YAP observed when cells are deprived of serum. This rescue of YAP regulation upon exogenous NF2 expression is observed in both *NF2* KO clone #1 (c) and clone #2 (d). **e,** Violin plot showing levels of nuclear YAP, as determined by immunofluorescence based images of cell monocultures, such as those shown in figure 3e, normalised to levels of cytoplasmic YAP (n = 8). **f,** Confocal based image acquired on the Opera Phenix Plus. Representative maximum projection images showing difference in YAP nuclear localisation between WT and *NF2* KO MeT‐5A cells. Cells were mixed before seeding, with *NF2* KO cells stained with CellTracker Red. Individual channels (taken from region within red dashed box) highlight the relative increase in nuclear YAP in *NF2* KO cells (diagonal arrows) relative to WT cells (arrowheads). Scale bar = 50 μm. **g,** Representative western blot (top) showing levels of LATS1/2 in MeT‐5A KO cells of different genotypes, with quantification of LATS2 (n = 3) below, with bars showing mean levels and error bars representing SD across replicates. LATS1 appears unchanged across all genotypes, while LATS2 is generally upregulated in both *NF2* and *BAP1* KO MeT‐5A. **h,** Bar‐plot shows qPCR quantification of *LATS2* expression relative to WT cells across *BAP1* KO and *YAP* KO MeT‐5A cells. Expression is shown as FC relative to WT MeT‐5A (n = 3). **i‐j,** Western blots show CRISPR‐mediated knockout of *YAP* in MeT‐5A cells (i), as well as *YAP* or *TAZ* double knockout (dKO) on *NF2* KO background MeT‐5A cells (j).

**Supplementary figure 4| The transcriptional effects of *BAP1* loss *in vitro* consistently and robustly mirrors mutation in patients**.
**a,** Heat‐map shows expression of 1,540 genes included in the combined NanoString PanCancer Progression and Immune Profiling nCounter panels across various KO MeT‐5A cell‐lines. Cells were analysed at steady‐state (‘+’) and after 3 hours of serum starvation (‘‐’). Dendrograms show the clustering of genotypes, highlighting similarities in transcriptional profiles observed in lines bearing the same KO. **b,** Heat‐map, as in figure 4d, shows the quantification of expression of genes found to be significantly differentially expressed in *BAP1* KO relative to WT MeT‐5A cells in patients from both cohorts grouped. No patients were excluded in this analysis, with mutation status for *BAP1* and Hippo‐associated genes annotated above. **c,** Violin plots show the GSVA scores in patients from both cohorts grouped, of genesets consisting of genes found to be dysregulated in BAP1 KO MeT‐5A cells. Genes found to be both up‐regulated (left) and down‐regulated (right) in vitro are similarly dysregulated in patients. **d,** Heatmap shows dysregulation of YAP/TAZ signature genes within *BAP1* KO MeT‐5A cells. Expression is shown across two distinct clones as mean log2FC relative to WT cells across biological replicates (n = 4). **e,** Scatter‐plots as in figure 4c‐d show correlation between gene dysregulation in *NF2* mutant patients from the Bueno *et al* cohort vs *NF2* KO MeT‐5A cells. Noise has been reduced by limiting analysis to those genes found to be significantly dysregulated in vitro. While some significant correlation exists in one *NF2* KO clone, this is not consistent, which suggests the same degree of conserved, broad transcriptomic dysregulation observed with *BAP1* disruption is not reproduced on loss of *NF2*. *P* values for (c) were determined via Kruskal‐Wallis tests, while correlation coefficients and *P* values for (d) were determined via the Spearman method, *P < 0.05.

**Supplementary figure 5| YAP‐TEAD coordinate regulation of expression of NF2 downstream targets**.
**a,** Bar‐plots show expression of putative NF2 target genes, as determined by qPCR. Expression is quantified in both *NF2* (red) and *YAP* (green) KO MeT‐5A cells, with expression plotted as fold‐change relative to WT MeT‐5A cells. In total, 10 dysregulated genes, as inferred from NanoString nCounter analysis, were assessed, comprising 4 up‐regulated (left) and 6 down‐regulated (right) genes. **b,** Scatter‐plot shows dysregulation of genes included in (a) in *NF2* KO cells plotted against *YAP* KO cells. A clear inverse correlation was observed between FCs relative to WT MeT‐5A in both *NF2* KO clone #1 (light red) and #2 (dark red). **c,** ChIP‐seq tracks, obtained using the Cistrome Data Browser, showing TEAD1 (top) and TEAD4 (bottom) co‐localisation at the most up‐ (*KISS1*, left) and down‐ (*CXCL8*, right) regulated NF2 genes. Co‐localisation was assessed in a number of publicly available cancer cell‐lines, comprising a range of different cancer‐types, including PM (MSTO‐211H, top track). TEAD peaks were observed at the promoter region of both genes (dark shading) across most of the cell‐lines included in analysis. Correlation coefficients and *P* values in (b) were determined by Pearson methods.
**Table 1| Lisa analysis of NF2 KO differentially expressed genes**.
Results from Lisa analysis^103^, showing the top 10 most significant P values across corresponding transcriptional regulators (including both transcription factors and chromatin regulators). The top 5 most significant samples, which consist of cell‐lines in which ChIP‐seq data corresponding to that regulator have been deposited in the Cistrome database, are included for each regulator.
