# Supplementary figures and images for: YAP/TAZ activation predicts clinical outcomes in mesothelioma and is conserved in in vitro model of driver mutations
Source: Clin Transl Med. 2023 Feb 5;13(2):e1190. doi: 10.1002/ctm2.1190 (PMC9899629; doi:10.1002/ctm2.1190)

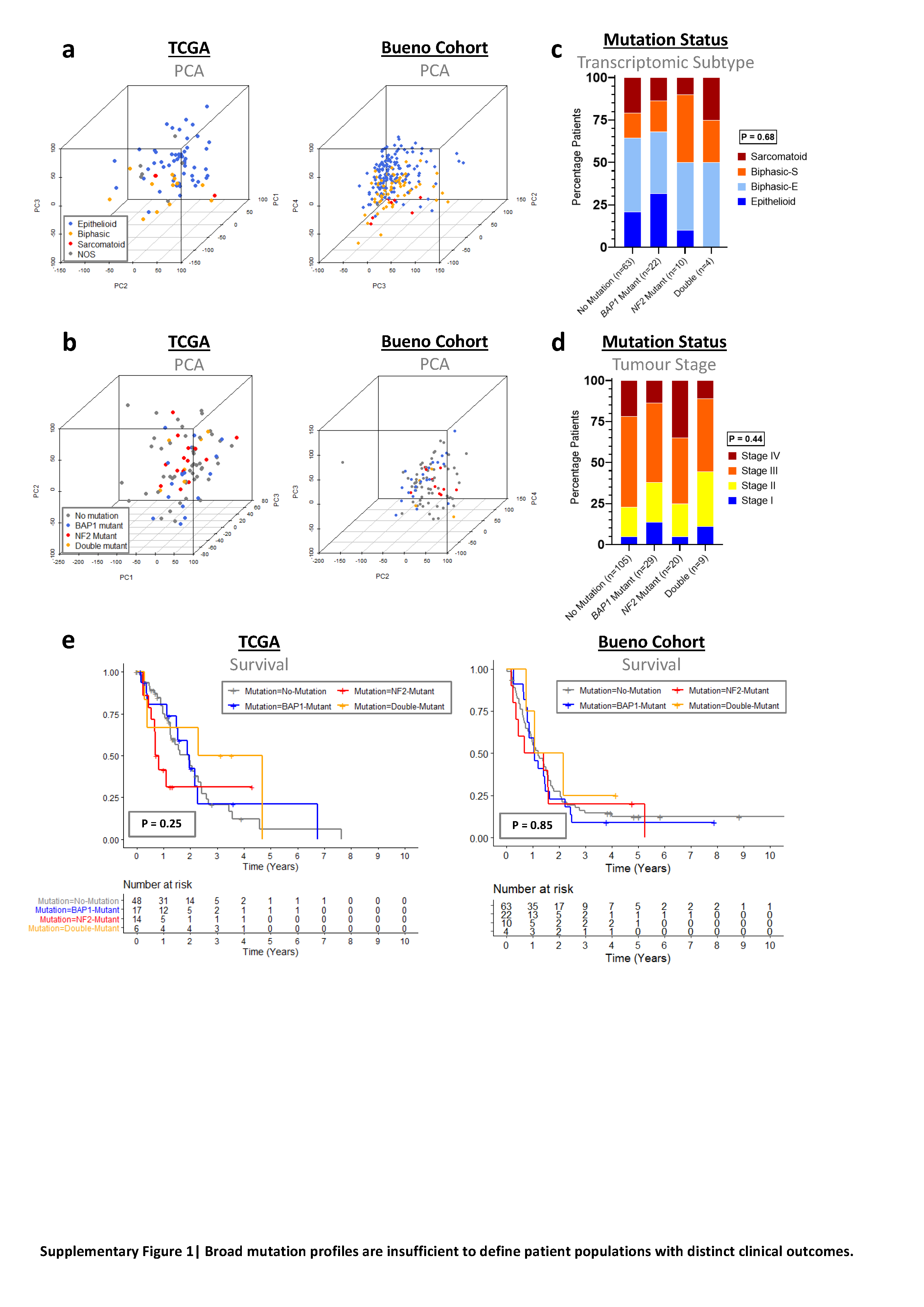

Supplement: Supplementary file 1 — Supplementary Figure 1| Broad mutation profiles are insufficient to define patient populations with distinct clinical outcomes. a, PCA plots displayed as in figure 1d, show PCs along different axes to highlight clusters. b, PCA plots, depicted in 3D, show patients coloured according to mutation status. Broad transcriptional profiling is insufficient to distinguish between the populations of patients categorised by mutation of BAP1 and NF2 in the TCGA (left; n = 86) and Bueno et al (right; 98) cohorts. c, Barplot shows the percentage of patients classified by consensus subtypes as generated in Bueno et al 16, with patients split according to mutation status of BAP1 and NF2. n = 98. d, Barplot, as in (c), highlights the absence of significant association between mutation status and T‐staging across the merged TCGA and Bueno et al datasets (n = 163). e, Kaplan‐Meier curves show overall survival of patients split according to mutation status, with patients categorised as exhibiting either NF2 or BAP1 mutations, as well as mutations in both or neither. No significant difference in survival across the patient populations is observed. P values for (c) and (d) determined via Fisher's exact test, while P values for (e) were calculated via log‐rank test. [file CTM2-13-e1190-s004.tiff]

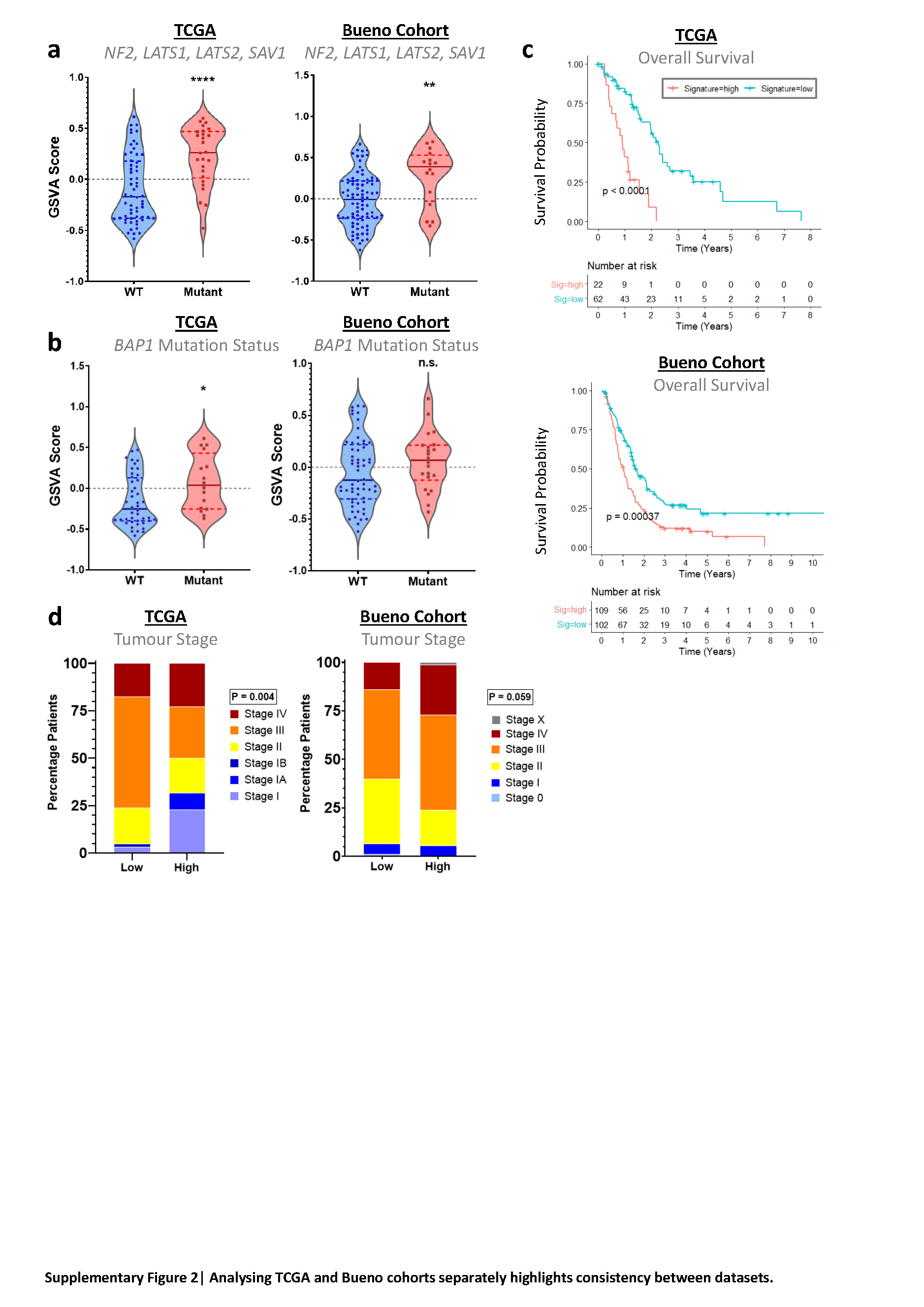

Supplement: Supplementary file 2 — Supplementary Figure 2| Analysing TCGA and Bueno cohorts separately highlights consistency between datasets. a, Violin plots show GSVA scores of YAP/TAZ signature gene expression as in figure 2a, with results split by dataset. A significant collective overexpression of this gene‐set is observed in patients with Hippo kinase cascade inactivating mutations in both TCGA (left; n = 86) and Bueno et al (right; n = 98) cohorts. b, Violin plots as in (a), show signature scores in patients split according to BAP1 mutation status, excluding patients harbouring Hippo pathway inactivating mutations. While there is an association between BAP1 mutation and expression of YAP/TAZ target genes, this is minor and only seen to be significant in the TCGA (left) and not Bueno et al (right) cohort. c, Kaplan‐Meier curves show overall survival of patients split according to YAP/TAZ signature thresholds. Patients classed as YAP/TAZ signature display a pronounced reduction in overall survival in both TCGA (top; HR = 4.4, n = 86, threshold at 74%) and Bueno et al (top; HR = 1.76, n = 211, threshold set at 52%) cohorts. d, Bar‐plots, as in figure 2e, show tumour stage in patients categorised as signature high/low. P values in (a) and (b) were determined by Mann‐Whitney U test, P values and hazard ratios for (c) were calculated via log‐rank test and Cox proportional hazard model respectively, while P values for (d) were calculated via Fisher's exact test. n.s. = Not significant, *P < 0.05, **P < 0.01, and ****P < 0.0001 relative to WT. [file CTM2-13-e1190-s002.tiff]

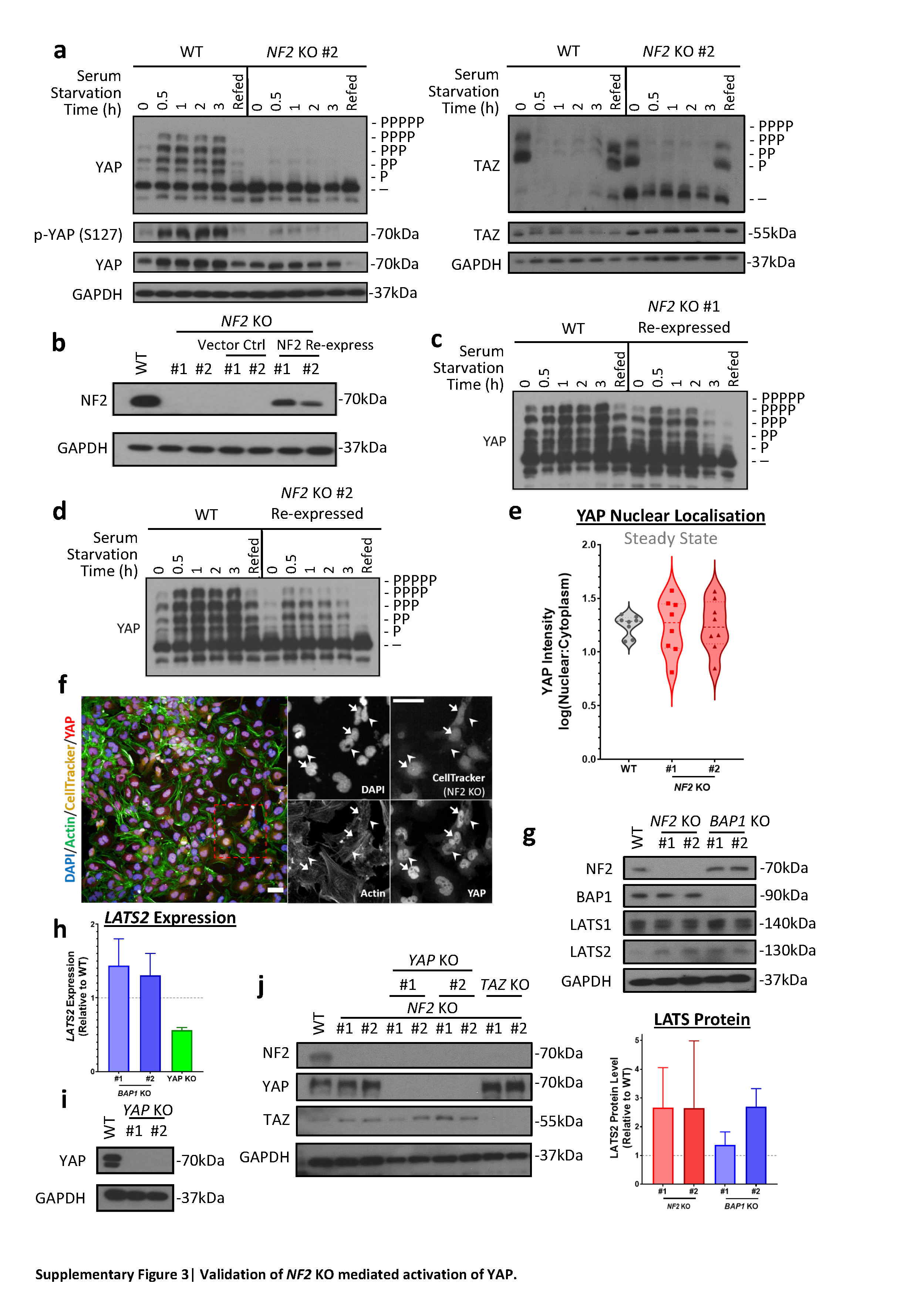

Supplement: Supplementary file 3 — Supplementary Figure 3| Validation of NF2 KO mediated activation of YAP. a, Phos‐tag based western blots (top), as in as in figure 3c, shows phosphorylation status of YAP (left) and TAZ (right) in response to serum starvation across a range of time‐points. Responses in WT MeT‐5A cells are compared to the second NF2 KO clone (#2), with a similar decreased sensitivity to starvation observed on NF2 loss in NF2 KO #1. A standard SDS‐gel based Western blot (bottom) is also shown with the same samples analysed, highlighting levels of phospho‐YAP (S127) and YAP (left), TAZ (right), together with GAPDH. b, Western blots showing NF2 expression levels in WT, NF2 KO, and re‐expression of NF2 in NF2 KO MeT‐5A clones. c‐d, Phos‐tag based western blots, as in (a), with NF2 re‐expressed in NF2 KO MeT‐5As. Response to serum starvation is restored upon NF2 re‐expression, with increased phosphorylation of YAP observed when cells are deprived of serum. This rescue of YAP regulation upon exogenous NF2 expression is observed in both NF2 KO clone #1 (c) and clone #2 (d). e, Violin plot showing levels of nuclear YAP, as determined by immunofluorescence based images of cell monocultures, such as those shown in figure 3e, normalised to levels of cytoplasmic YAP (n = 8). f, Confocal based image acquired on the Opera Phenix Plus. Representative maximum projection images showing difference in YAP nuclear localisation between WT and NF2 KO MeT‐5A cells. Cells were mixed before seeding, with NF2 KO cells stained with CellTracker Red. Individual channels (taken from region within red dashed box) highlight the relative increase in nuclear YAP in NF2 KO cells (diagonal arrows) relative to WT cells (arrowheads). Scale bar = 50 μm. g, Representative western blot (top) showing levels of LATS1/2 in MeT‐5A KO cells of different genotypes, with quantification of LATS2 (n = 3) below, with bars showing mean levels and error bars representing SD across replicates. LATS1 appears unchanged across all genotype [file CTM2-13-e1190-s003.tiff]

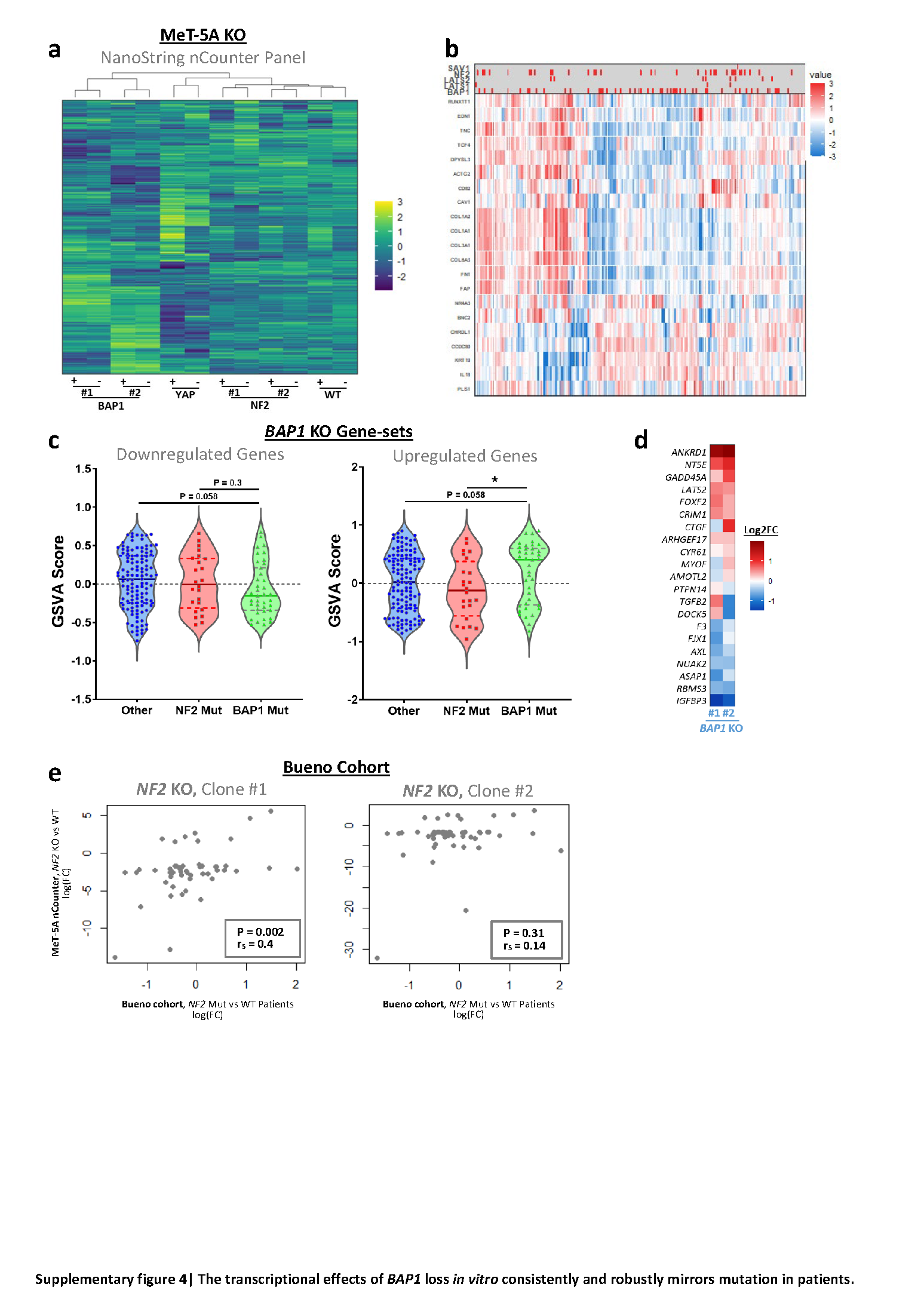

Supplement: Supplementary file 4 — Supplementary figure 4| The transcriptional effects of BAP1 loss in vitro consistently and robustly mirrors mutation in patients. a, Heat‐map shows expression of 1,540 genes included in the combined NanoString PanCancer Progression and Immune Profiling nCounter panels across various KO MeT‐5A cell‐lines. Cells were analysed at steady‐state (‘+’) and after 3 hours of serum starvation (‘‐’). Dendrograms show the clustering of genotypes, highlighting similarities in transcriptional profiles observed in lines bearing the same KO. b, Heat‐map, as in figure 4d, shows the quantification of expression of genes found to be significantly differentially expressed in BAP1 KO relative to WT MeT‐5A cells in patients from both cohorts grouped. No patients were excluded in this analysis, with mutation status for BAP1 and Hippo‐associated genes annotated above. c, Violin plots show the GSVA scores in patients from both cohorts grouped, of genesets consisting of genes found to be dysregulated in BAP1 KO MeT‐5A cells. Genes found to be both up‐regulated (left) and down‐regulated (right) in vitro are similarly dysregulated in patients. d, Heatmap shows dysregulation of YAP/TAZ signature genes within BAP1 KO MeT‐5A cells. Expression is shown across two distinct clones as mean log2FC relative to WT cells across biological replicates (n = 4). e, Scatter‐plots as in figure 4c‐d show correlation between gene dysregulation in NF2 mutant patients from the Bueno et al cohort vs NF2 KO MeT‐5A cells. Noise has been reduced by limiting analysis to those genes found to be significantly dysregulated in vitro. While some significant correlation exists in one NF2 KO clone, this is not consistent, which suggests the same degree of conserved, broad transcriptomic dysregulation observed with BAP1 disruption is not reproduced on loss of NF2. P values for (c) were determined via Kruskal‐Wallis tests, while correlation coefficients and P values for (d) were determined via the Spearman method, *P < 0.05. [file CTM2-13-e1190-s001.tiff]

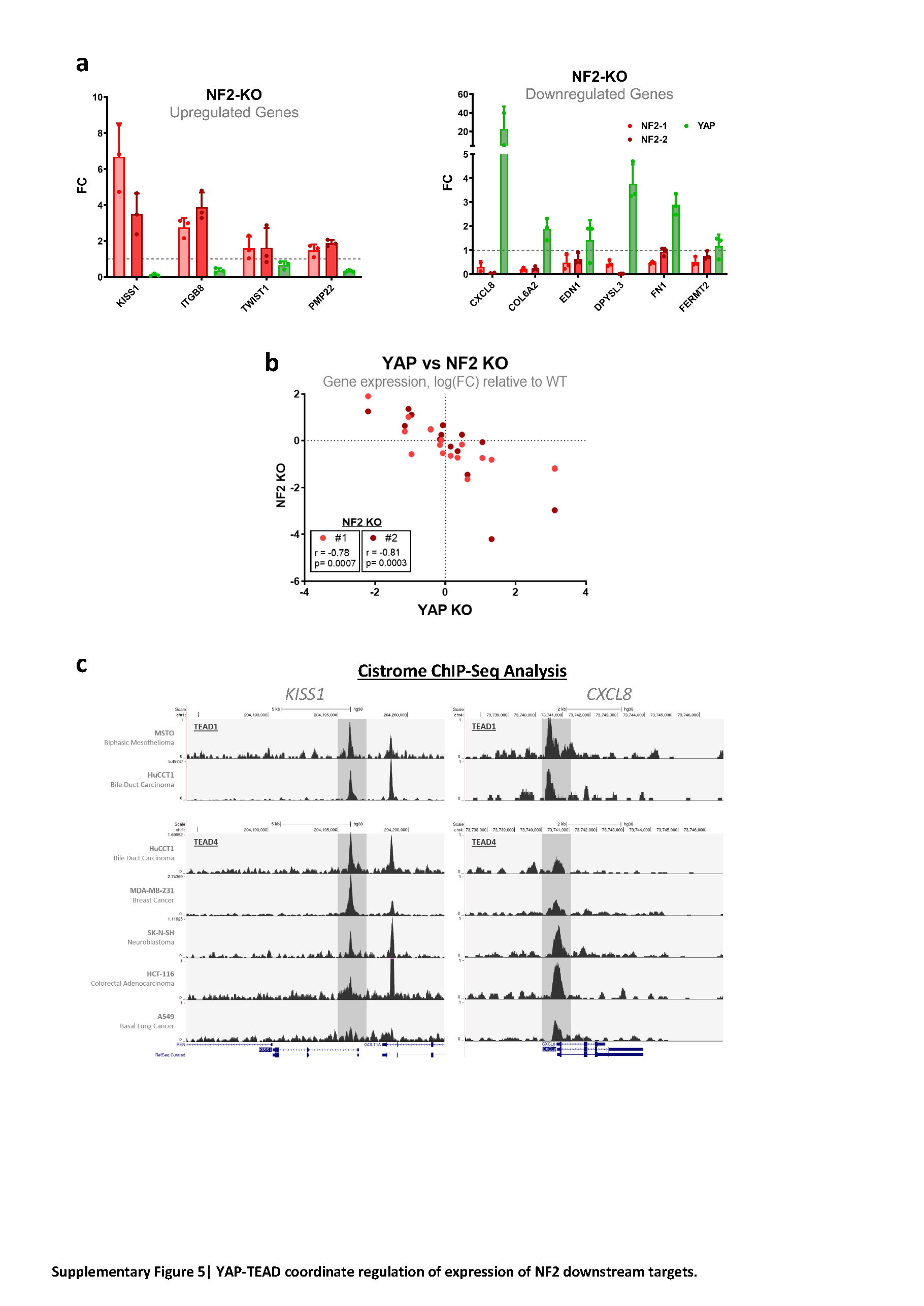

Supplement: Supplementary file 5 — Supplementary figure 5| YAP‐TEAD coordinate regulation of expression of NF2 downstream targets. a, Bar‐plots show expression of putative NF2 target genes, as determined by qPCR. Expression is quantified in both NF2 (red) and YAP (green) KO MeT‐5A cells, with expression plotted as fold‐change relative to WT MeT‐5A cells. In total, 10 dysregulated genes, as inferred from NanoString nCounter analysis, were assessed, comprising 4 up‐regulated (left) and 6 down‐regulated (right) genes. b, Scatter‐plot shows dysregulation of genes included in (a) in NF2 KO cells plotted against YAP KO cells. A clear inverse correlation was observed between FCs relative to WT MeT‐5A in both NF2 KO clone #1 (light red) and #2 (dark red). c, ChIP‐seq tracks, obtained using the Cistrome Data Browser, showing TEAD1 (top) and TEAD4 (bottom) co‐localisation at the most up‐ (KISS1, left) and down‐ (CXCL8, right) regulated NF2 genes. Co‐localisation was assessed in a number of publicly available cancer cell‐lines, comprising a range of different cancer‐types, including PM (MSTO‐211H, top track). TEAD peaks were observed at the promoter region of both genes (dark shading) across most of the cell‐lines included in analysis. Correlation coefficients and P values in (b) were determined by Pearson methods. Table 1| Lisa analysis of NF2 KO differentially expressed genes. Results from Lisa analysis103, showing the top 10 most significant P values across corresponding transcriptional regulators (including both transcription factors and chromatin regulators). The top 5 most significant samples, which consist of cell‐lines in which ChIP‐seq data corresponding to that regulator have been deposited in the Cistrome database, are included for each regulator. [file CTM2-13-e1190-s005.tiff]
